# Molecular characterization, expression and functional analysis of acyl-CoA-binding protein gene family in maize (*Zea mays*)

**DOI:** 10.1186/s12870-021-02863-4

**Published:** 2021-02-15

**Authors:** Jiantang Zhu, Weijun Li, Yuanyuan Zhou, Laming Pei, Jiajia Liu, Xinyao Xia, Ronghui Che, Hui Li

**Affiliations:** School of Biological Science and Technology, University of Jinan, Jinan, 250022 China

**Keywords:** *Zea mays*, Acyl-CoA binding protein (ACBP), Evolution, Subcellular localization, Expression profiles, Stress

## Abstract

**Background:**

Acyl-CoA-binding proteins (ACBPs) possess a conserved acyl-CoA-binding (ACB) domain that facilitates binding to acyl-CoA esters and trafficking in eukaryotic cells. Although the various functions of ACBP have been characterized in several plant species, their structure, molecular evolution, expression profile, and function have not been fully elucidated in *Zea mays* L.

**Results:**

Genome-wide analysis identified nine *ZmACBP* genes in *Z. mays*, which could be divided into four distinct classes (class I, class II, class III, and class IV) via construction of a phylogenetic tree that included 48 *ACBP* genes from six different plant species. Transient expression of a ZmACBP-GFP fusion protein in tobacco (*Nicotiana tabacum*) epidermal cells revealed that ZmACBPs localized to multiple different locations. Analyses of expression profiles revealed that *ZmACBPs* exhibited temporal and spatial expression changes during abiotic and biotic stresses. Eight of the nine *ZmACBP* genes were also found to have significant association with agronomic traits in a panel of 500 maize inbred lines. The heterologous constitutive expression of *ZmACBP1* and *ZmACBP3* in Arabidopsis enhanced the resistance of these plants to salinity and drought stress, possibly through alterations in the level of lipid metabolic and stress-responsive genes.

**Conclusion:**

The *ACBP* gene family was highly conserved across different plant species. *ZmACBP* genes had clear tissue and organ expression specificity and were responsive to both biotic and abiotic stresses, suggesting their roles in plant growth and stress resistance.

**Supplementary Information:**

The online version contains supplementary material available at 10.1186/s12870-021-02863-4.

## Background

Lipids provide the structural basis for cell membranes and energy for metabolic processes, and also serve as signaling molecules during environmental stress responses in plants [1]. Lipids and their derivatives are transported to different locations in the cell to function properly by various proteins, such as the lipid-transfer proteins (LTPs) and the acyl-CoA-binding proteins (ACBPs) [2, 3]. During fatty acid synthesis (FAS) in eukaryotes, the majority of long-chain fatty acids (LCFA, e.g. C16 and C18) are first converted into acyl-CoAs by long-chain acyl-CoA synthetase (LACS) in the plastid [4]. Afterwards, the majority of the resulting products dissociate from the acyl carrier proteins via thioesterases, and are converted to 16:0-CoA, 18:0-CoA, and 18:1-CoA esters in the cytosol, following transport to the endoplasmic reticulum (ER) [5]. ACBPs contain an acyl-CoA-binding (ACB) domain that is able to bind to long-chain acyl-CoA esters in order to facilitate the transport of acyl-CoAs and fatty acids from chloroplast or plastid to the ER [6].

ACBPs have been extensively characterized in eukaryotes, with a number of important biological functions identified [7–9]. For example, yeast ACBPs play important roles in the assembly of the plasma membrane, and mutations in these genes significantly impact a number of different pathways, such as fatty acid biosynthesis, glycerol metabolism, and stress response [10, 11]. ACBPs are also essential for the biosynthesis of membrane, the regulation of enzyme activities, the lipid metabolism, and the response to stress of plants [12]. Based on their domain architecture, plant ACBPs can be divided into four classes: class I (small ACBPs) contains only one ACB domain; class II (ankyrin-ACBPs) additionally contains ankyrin repeats in their C-terminals; class III (large-ACBPs) is large proteins with C-terminal ACB domains and class IV (kelch-ACBPs) is multi-domain proteins containing C-terminal kelch motifs [13–15].

In Arabidopsis, there are six genes encoding ACBPs (designated AtACBP1–6), which have their own characteristics in structure, expression, subcellular location, acyl-coA ester affinities and functions [16]. AtACBP1 (338 amino acids, 37.5 kDa) and AtACBP2 (355 amino acids, 38.5 kDa), belonging to class II, are located in the ER and plasma membrane [17], and both of these proteins can bind C18:2-CoA, C18:3-CoA esters, phosphatidylcholine (PC), and phosphatidic acid (PA) [18]. *AtACBP1* is highly expressed in reproductive organs such as seeds and siliques, while *AtACBP2* is expressed in vegetative organs such as roots and stems [18]. AtACBP1 and AtACBP2 are known to function in maintaining the membrane-associated acyl pool, and are also involved in various stress responses, such as freezing, drought, Cd (II) and oxidative stress [17–19]. AtACBP3 (362 amino acids, 39.3 kDa), being class III ACBPs, is a multi-localized protein, and found in membrane, extracellular and apoplast [6]. AtACBP3 can bind arachidonyl-CoA (C20:4), PC, and phosphatidylethanolamine (PE), and is mainly found in siliques and young shoots [16]. AtACBP3 plays an important role in maintaining normal lipid balance, and is involved in a number of biological processes, including the regulation of leaf senescence, response to pathogen infection and others [20]. Class IV ACBPs include AtACBP4 (668 amino acids, 73.3 kDa) and AtACBP5 (648 amino acids, 71 kDa), which are both cytosolic proteins and can bind C18:1-CoA and PC [21]. *AtACBP4* is strongly expressed in roots, while the expression level of *AtACBP5* is higher in young shoots and mature leaves [21]. AtACBP4 and AtACBP5 are suggested to act on pollen development and defense reaction by impacting lipid metabolism [22, 23]. AtACBP6 (92 amino acids, 10.4 kDa), being the smallest ACBPs, was found to be localized to the cytosol and has high affinity for 16:0-CoA and 18:2-CoA [16]. AtACBP6 is found in all plant organs, and has been implicated in intracellular binding and trafficking of phosphatidylcholine in plant phospholipid metabolism [16, 24]. There are also six ACBPs in *Oryza sativa*, and OsACBPs display difference in its affinity for substrates, tissue expression, and response to stress compared with AtACBPs. For example, OsACBP1 and OsACBP4 bind C18:1-CoA and C18:2-CoA, respectively, whereas other OsACBPs bind C18:3-CoA [13]. Previous work has shown that the expression levels of *OsACBP1*, *OsACBP2*, and *OsACBP3* were not affected under drought and high salinity treatments, while *OsACBP4* and *OsACBP5* were induced under these conditions [13].

Although ACBPs have been investigated for some plant species, such as Arabidopsis, rice, and *Brassica napus*, no reports to date have comprehensively analyzed the maize ACBP family. In this study, we reported the phylogenetic evolution, the expression profiles of *ACBP* genes in different tissues or stress treatments, and association with agronomic traits in maize. These findings are helpful to further study the biological functions of ZmACBPs in development and stress responses in maize.

## Results

### Distribution of ACBP family genes in different plant species

**Fig. 1:**
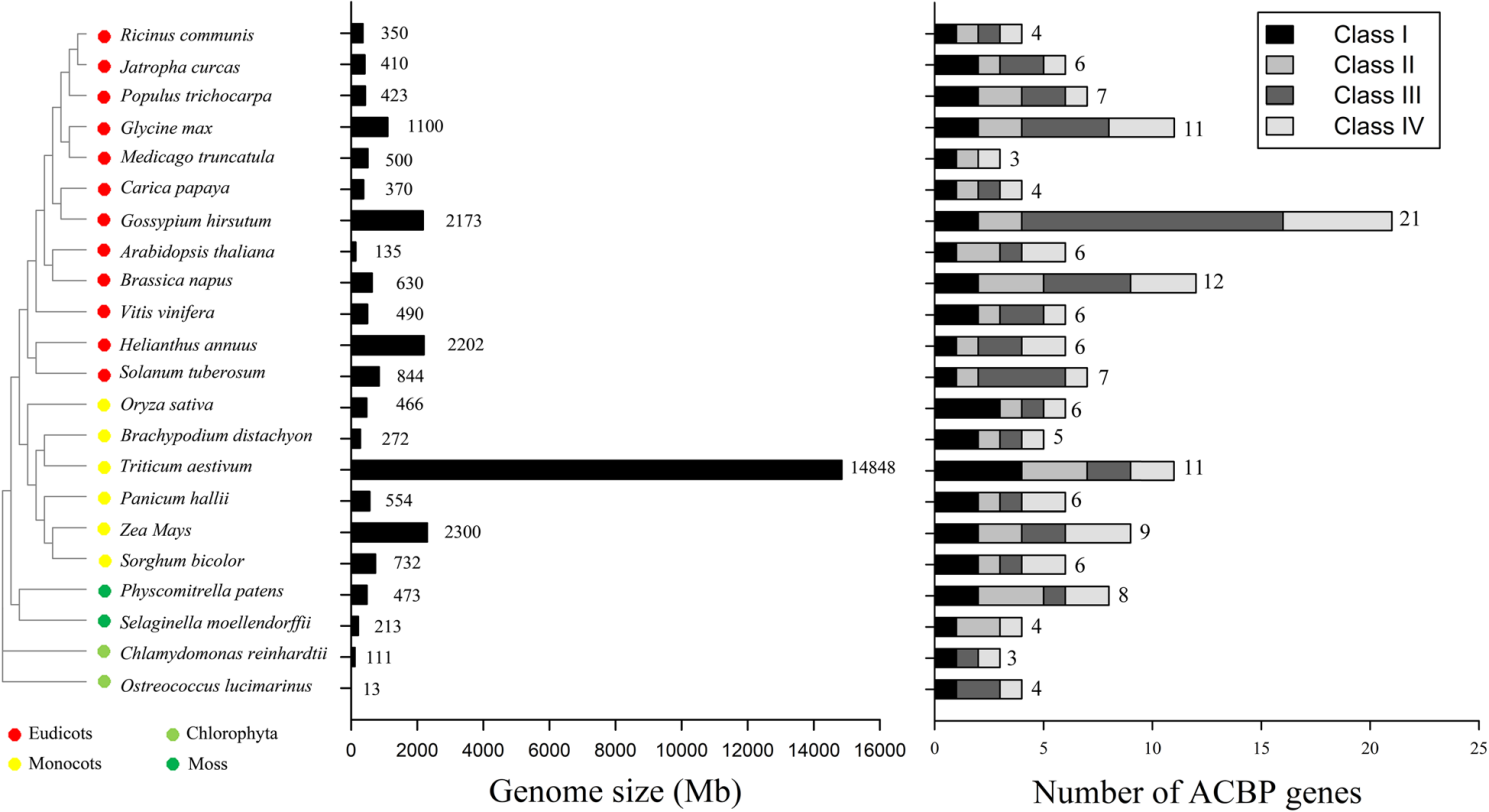
The distribution of the ACBP family genes in different plant species. The left is the phylogenetic tree ACBP from 22 plant species; the middle is the genome size of 22 plant species; the right is the number of *ACBP* genes in 22 plant species. Dots of different colors represent the different species (Red, Eudicots; light green, Chlorophyta; yellow, Monocots; Green, Moss)

To determine the distribution of ACBPs in the plant kingdom, the amino acid sequences of ACBPs from Arabidopsis were utilized to search the Phytozome database, and the sequences of genes encoding ACBPs in 22 representative species were obtained (Fig. 1). Although ACBPs are ubiquitous in the plant kingdom, their number and type varied significantly (Fig. 1). In Chlorophyta, which branched off before the evolution of land plants, *Ostreococcus lucimarinus* and *Chlamydomonas reinhardtii* were found to contain four and three *ACBP* genes, respectively (Fig. 1). There were four *ACBP* genes in *Selaginella moellendorffii*, which belongs to the lycophyte clade and is the origin of seed plants. Additionally, the number of *ACBP* genes were increased to eight in the moss *Physcomitrella patens*, which is thought to be the progenitor of vascular plants (Fig. 1). Although monocots evolved much more recently than mosses, the gene numbers of the ACBP decreased to five in *Brachypodium distachyon*, six in rice, six in *Sorghum bicolor* (Fig. 1). However, there were nine *ACBP* genes in maize and 11 in *Triticum aestivum* (Fig. 1). Interestingly, it appeared that there was no significant relationship between the number of *ACBP* genes and the size of the species’ genome. For example, the maize genome is eight-fold larger than that of *B. distachyon*, but maize only contains four more *ACBP* genes (Fig. 1). Compared with monocot plants, the number of *ACBP* genes in eudicot plants varied greatly. For example, there were only three *ACBP* genes in the *Medicago truncatula* genome, while 11 *ACBP* genes were found in *Glycine max* (Fig. 1). Similarly, there were only six *ACBP* genes in Arabidopsis, but up to 12 in *B. napus* (Fig. 1).

### Identification and characterization of *ACBP* genes in maize

A total of nine putative ZmACBP sequences were identified in the Maize Genome Database (https://www.maizegdb.org), which were then termed ZmACBP1–9. The general features of the *ZmACBP* gene family members, including identifier (ID), gene name, open reading frame size (ORF), exon number, amino acid length, molecular weight (MW), and isoelectric point (PI) of protein are shown in Table 1. The number of amino acids (aa) in ZmACBPs varied from 89 aa (ZmACBP1) to 783 aa (ZmACBP9). Similar to *A. thaliana*, the conserved ACB domain was located at the N-terminal region in most ZmACBPs, although ZmACBP5 and ZmACBP6 both contained a C-terminal ACB domain (Additional file 1: Fig. S1a). The nine ZmACBPs were divided into four classes based on their domain structures (Additional file 1: Fig. S1a). Class I had two members, ZmACBP1 and ZmACBP2, and both of them contained only one ACB domain (Additional file 1: Fig. S1a). ZmACBP3 and ZmACBP4 possessed an ACB domain and ankyrin repeats and belonged to class II. Class III, being a large ACBP class, had ZmACBP5 and ZmACBP6 (Additional file 1: Fig. S1a). ZmACBP7, ZmACBP8, and ZmACBP9 possessed an ACB domain and a kelch motif, respectively, and belonged to class IV (Additional file 1: Fig. S1a). The nine *ZmACBP* genes were located on five different chromosomes (Table 1, Additional file 1: Fig. S1b). Three *ZmACBP* genes were located on chromosome 1, two *ZmACBP* genes were found on chromosomes 5 or 10, and only one *ZmACBP* gene was located on chromosomes 2 and 9 (Additional file 1: Fig. S1b). Examination of the location of each *ZmACBP* gene revealed that *ZmACBP7* and *ZmACBP8* were arranged in a tandem duplication (Additional file 1: Fig. S1b). The syntenic maps of maize compared with rice were analyzed, which revealed that 7 out of the 9 *ZmACBP* genes had collinear genes in rice (Additional file 1: Fig. S1c; Additional file 2: Table S1). This indicated that the collinear pairs were likely derived from a common ancestor.

**Table 1:** General information of *ACBP* genes in maize. ID: identifier; ORF: open reading frame; PI: isoelectric point; MW: molecular weight; aa: amino acid

Gene ID	Name	Chr.	ORF (bp)	Exon no.	Protein (aa)	PI	MW (KD)
GRMZM2G079908 (Zm00001d024518)	*ZmACBP1*	10	270	4	89	4.99	10.13
GRMZM2G344634 (Zm00001d045539)	*ZmACBP2*	9	276	3	91	5.83	9.97
GRMZM2G049495 (Zm00001d001798)	*ZmACBP3*	2	987	6	328	4.60	34.79
GRMZM2G173636 (Zm00001d026639)	*ZmACBP4*	10	987	6	328	4.59	35.12
GRMZM2G108138 (Zm00001d028397)	*ZmACBP5*	1	1635	5	544	4.15	57.93
GRMZM2G060781 (Zm00001d015957)	*ZmACBP6*	5	924	1	307	4.57	35.23
GRMZM2G053803 (Zm00001d034758)	*ZmACBP7*	1	1980	18	659	5.11	72.06
GRMZM2G351160 (Zm00001d034759)	*ZmACBP8*	1	1929	18	642	5.18	70.33
GRMZM2G326195 (Zm00001d012892)	*ZmACBP9*	5	2352	18	783	5.37	85.24

### Phylogenetic relationship, gene structure and conserved motifs of *ZmACBP* genes in maize

**Fig. 2:**
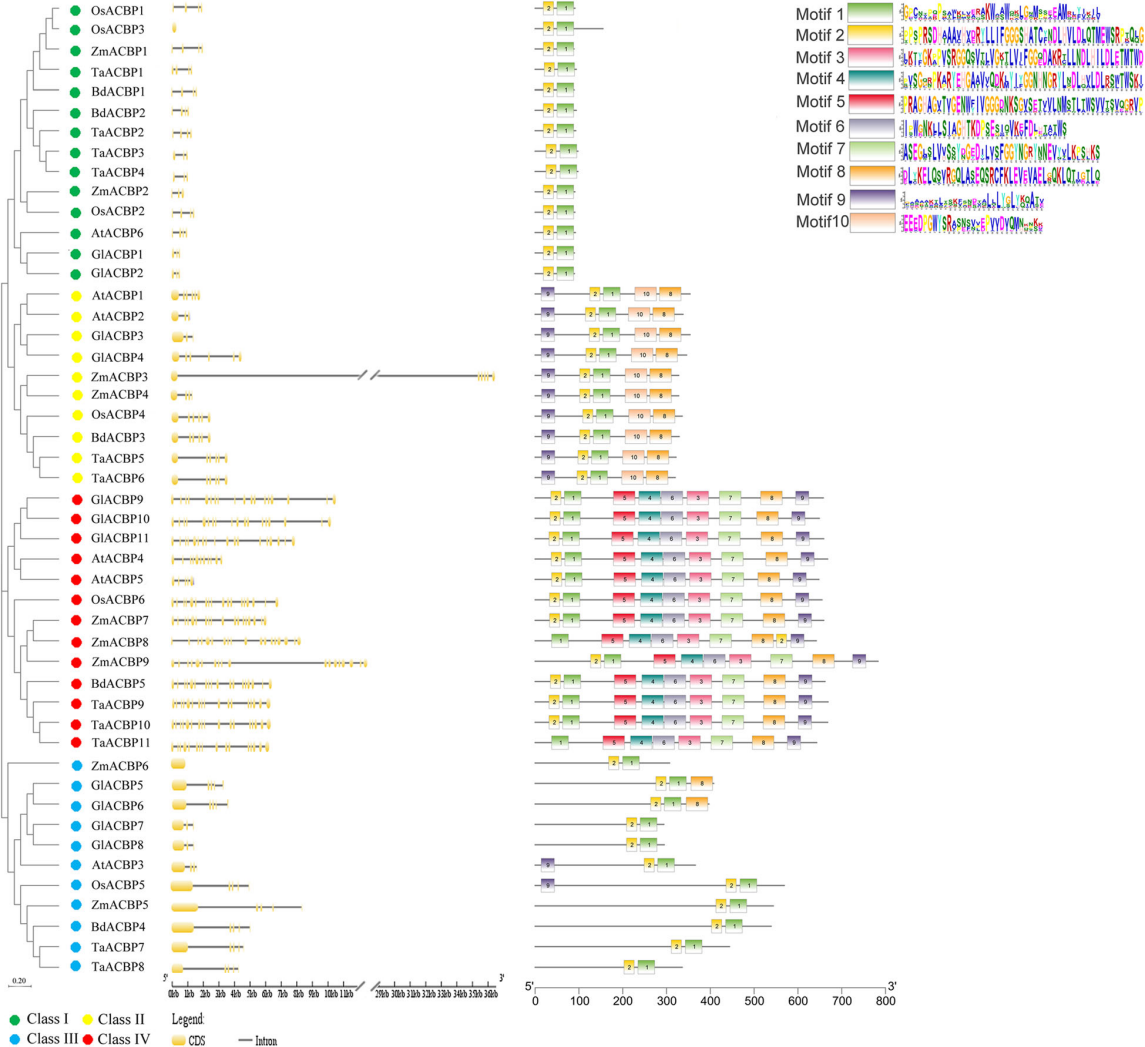
The phylogenic relationships, gene structures, and conserved motifs of maize ACBP family genes. The phylogenetic tree was constructed using MEGA 7.1 software by the Neighbor-Joining method with 1000 bootstrap replicates. The different *ACBP* classes were marked with dots of different colors: green, class I; yellow, class II; blue, class III; red, class IV. The yellow boxes and grey lines represent exons and introns, respectively. The wide boxes of different colors represent different conserved motifs. The scales were referred to the lengths of exons, introns and motifs, respectively

To analyze the evolutionary relationships among the ACBPs of different species, the sequences of nine ZmACBPs, six AtACBPs, six OsACBPs, 11 TaACBPs, five BdACBPs, and 11 GlACBPs were aligned, and the resulting alignment was utilized to generate an unrooted phylogenetic tree using the Neighbor-Joining method. A total of 48 ACBP sequences, including nine ZmACBPs, were sorted into four ACBP classes (Fig. 2). The phylogenetic tree clearly showed that all ZmACBPs were more likely to form closer clusters with the ACBPs from closely related species, such as rice, *B. distachyon*, and *T. aestivum*. Conversely, ACBPs from the more distantly related species formed farther cluster branches (Fig. 2).

Analysis of the exon/intron structures of the *ZmACBP* genes revealed that the number of exons significantly varied within the family, mostly ranging from three to six, whereas *ZmACBP7/− 8/− 9* had the greatest number of exons (up to 18 exons, Fig. 2). Interestingly, a similar gene structure was found within each class. For example, most of the *ACBPs* in class I contained four exons, while most class II and class III *ACBPs* contained seven. The vast majority of *ACBPs* in class IV, on the other hand, had 18 exons (Fig. 2). In addition, a total of 10 conserved motifs were identified in ACBPs using the online MEME tool, and all of the ACBP proteins had two different conserved motifs (motif 1 and 2), which were contained within the ACB domain (Fig. 2). The ACBPs in class II had two motifs (8 and 10) in their C-terminal regions, which corresponded to ankyrin repeats. The class IV ACBPs contained multiple motifs (3, 4, 5, 6, and 7), corresponding to C-terminal kelch domains (Fig. 2). It is worth noting that ACBPs clustered in the same clade had similar domain architectures, which suggested that ACBP was highly conserved during evolution.

### *Cis*-regulatory elements in the promoters of *ZmACBP* genes

**Fig. 3:**
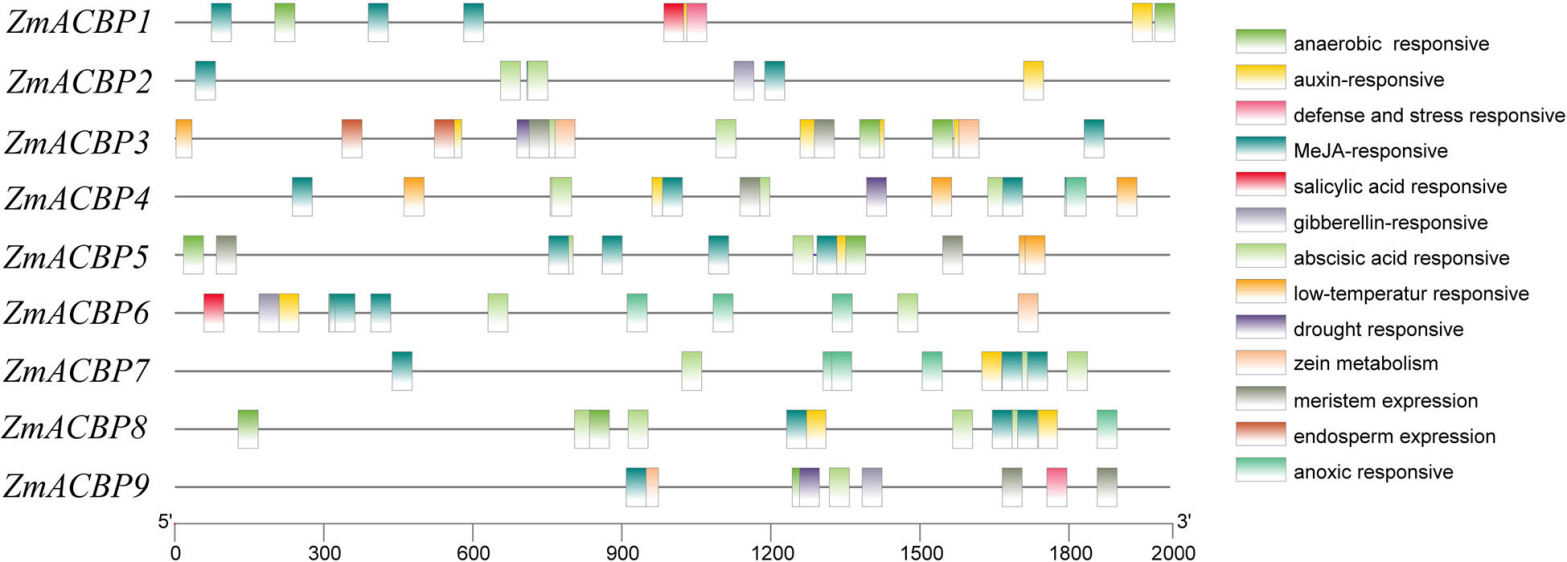
Putative regulatory *cis*-elements in maize ACBP family gene promoters. Boxes of different colors represent different *cis*-elements

To further explore the function and the regulation patterns of *ZmACBP* genes, the *cis*-acting regulatory elements at the regions of 1500 bp upstream from the initiation codons of nine *ZmACBP* genomic sequences were searched in the Plant CARE database. Multiple conserved *cis*-regulatory elements were found to reside in the *ZmACBP* promoters, with significant variations found among the different members (Fig. 3, Additional file 3: Table S2). The *cis*-acting regulatory elements may be involved into numerous physiological processes, for example, development-related, hormonal responses, and environment responses. A meristem expression element was found in the promoter regions of all nine *ZmACBP* genes, suggesting that the entire family may play a role in differentiation. Many *cis*-elements involved in hormone responses, including abscisic acid responsive elements (ABRE), MeJA-responsive elements (TGACG-motif and CGTCA-motif), salicylic acid responsive elements (TCA-element and SARE), auxin-responsive elements (TGA-element and AuxRR-core), and gibberellin responsive elements (TATC-box, P-box, and GARE-motif), were found within most *ZmABCP* gene promoters (Fig. 3), suggesting that *ZmABCP* genes may be involved in growth and development. In addition, some *cis*-elements thought to be involved in environmental stress responses, such as low-temperature responsive element (LTR), drought-inducibility element (MBS), anaerobic induction responsive element (ARE), and defense and stress responsive element (AT-rich and TC-rich repeats), were found in a few of the *ZmACBP* gene promoters. For example, a *cis*-element involved in low-temperature responsiveness was found in the promoters of *ZmACBP4* and *ZmACBP5* (Fig. 3), while defense and stress responsive elements were found in the promoters of *ZmACBP1* and *ZmACBP9* (Fig. 3).

### Subcellular localization of ZmACBPs

**Fig. 4:**
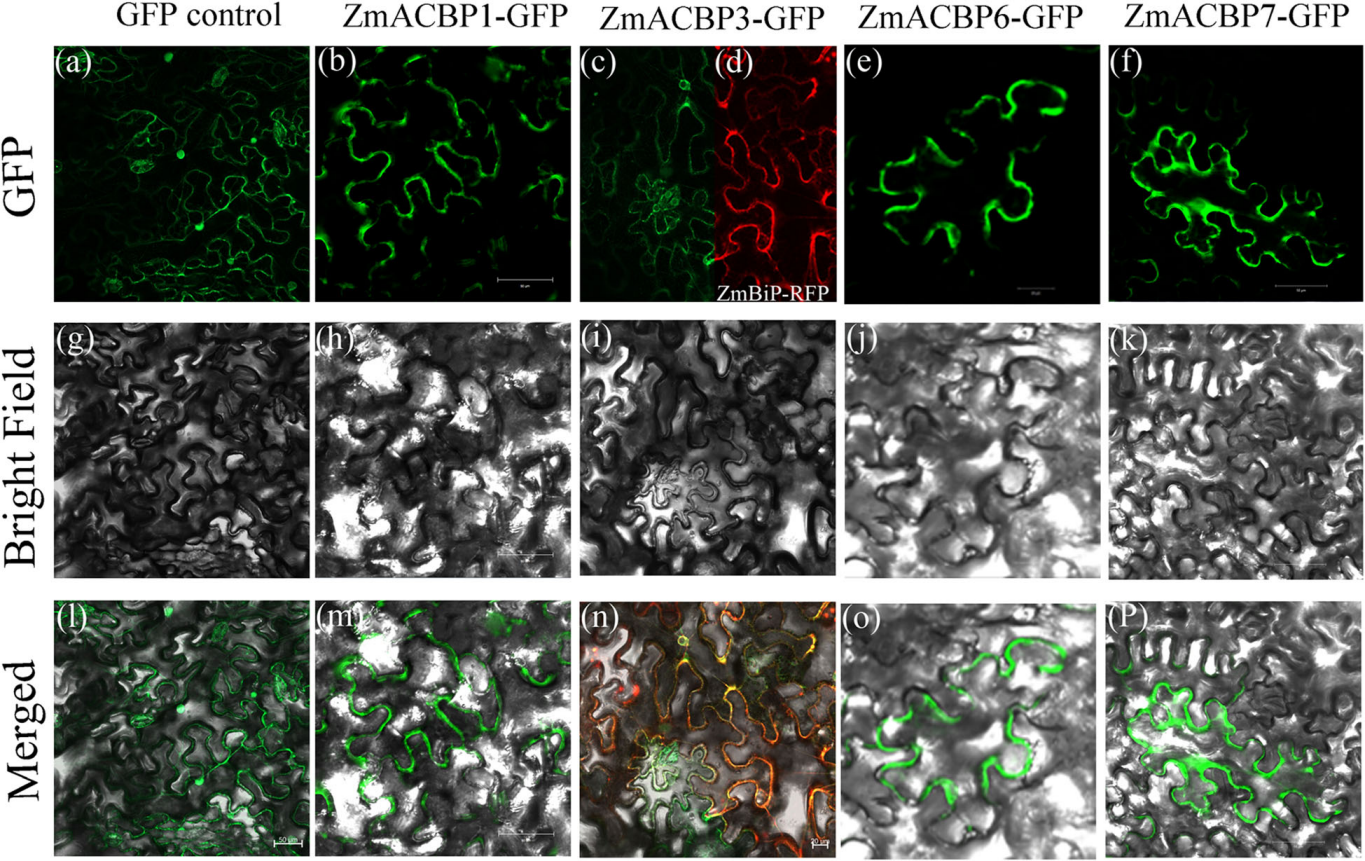
Subcellular localizations of ZmACBPs in tobacco leaf epidermal cells by confocal laser-scanning microscopy. **a-f** ZmACBPs-GFP fusion protein; **g-k** bright field; **l-p** Merged image of bright field with GFP fluorescence; **d** ER marker, ZmBiP-RFP. GFP control, *35S*::GFP from pBI-eGFP vector control

One randomly selected ZmACBP from each of the four classes was attached to a GFP tag and driven by the CaMV *35S* promoter. These constructs were then transiently expressed in *Nicotiana benthamiana* leaf epidermal cells to determine their subcellular localization. In *N. benthamiana* leaf epidermal cells, the fluorescence of the GFP control was observed in both the nucleus and cytoplasm (Fig. 4a). The GFP fluorescent signals of ZmACBP1::GFP were only found in the cytoplasm and were not detected at all in the nucleus (Fig. 4b), indicating that this protein might be localized to the cytosol. To further confirm the subcellular localization of ZmACBP1, *35S*::ZmACBP1::GFP was transiently expressed in *A. thaliana* mesophyll protoplasts, with a similar cytoplasm-specific signal detected in this system (Additional file 4: Fig. S2). The GFP fluorescent signals of ZmACBP3::GFP were distributed at the ER surrounding the nuclei (Fig. 4c). To confirm the ER localization of this protein, ZmACBP3::GFP was co-transfected into *N. benthamiana* leaf epidermal cells with the ER marker ZmBiP-RFP (Fig. 4d). The GFP (Fig. 4c) and RFP (Fig. 4d) signals were found to overlap (Fig. 4n), indicating that ZmACBP3::GFP is an ER-associated protein. The distributions of ZmACBP6::GFP and ZmACBP7::GFP were similar to the GFP control in tobacco leaf epidermal cells (Fig. 4e and f), while disappearing in nuclear localization. These results were consistent with those seen in Arabidopsis leaf protoplasts (Additional file 4: Fig. S2), indicating that ZmACBP6 and ZmACBP7 were targeted to the cytosol and plasma membrane.

### Expression profiles of *ZmACBP* in different organs and developmental stages

**Fig. 5:**
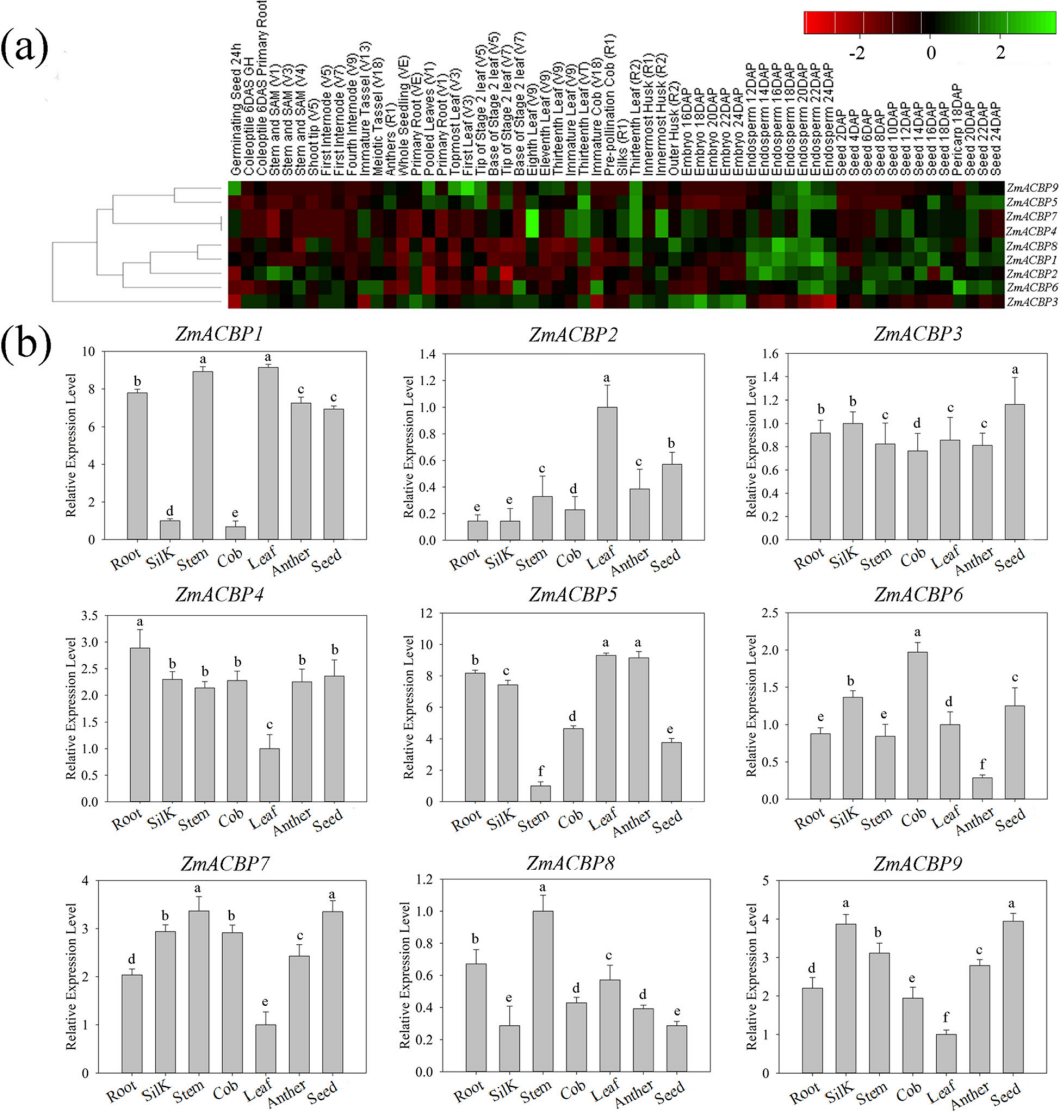
Tissue expression patterns of ACBP family genes in *Zea mays*. **a** Hierarchical clustering and classification of the nine *ZmACBP* genes based on RNA-seq data during development stages. **b** Tissue-specific expression patterns of the nine *ZmACBP* genes were in maize root, stem, leaf, silk, immature cob, anther, and kernel (10 DAP) by using Quantitative real-time PCR. The expression levels were normalized to expression levels of maize TUB-ribosylation factor. Data represent mean ± SE of three replicates. Different letters indicate values which departed significantly at the 0.01 among tissues

To analyze the expression profiles of nine *ZmACBP* genes during maize development, their transcriptional patterns were assessed basing on the microarray data (Maize eFP Browser, http://bar.utoronto.ca/efp_maize/cgi-bin/efpWeb.cgi?dataSource=Sekhon_et_al_Atlas) in 60 different tissues or developmental stages in maize. The heat map of microarray data shown that the expression patterns of the *ZmACBPs* could be classified into four main clusters (Fig. 5a). Cluster I included *ZmACBP5* and *ZmACBP9*, which were highly expressed in the thirteenth leaf and endosperm (18 ~ 22 DAP, days after pollination) and were much lower in other tissues (Fig. 5a). Cluster II contained two genes, *ZmACBP4* and *ZmACBP7*, which were highly expressed in the eighth and thirteenth leaves (Fig. 5a). The cluster III genes, *ZmACBP1*, *ZmACBP2*, *ZmACBP6*, and *ZmACBP8*, were expressed highly in the endosperm (12 ~ 24 DAP) and seed (Fig. 5a). *ZmACBP3*, belonging to Cluster IV, was highly expressed in the embryo (18 ~ 22 DAP) (Fig. 5a).

qRT-PCR was used to validate the expression levels of the nine *ZmACBP* genes in root, stem, leaf, silk, immature cob, anther, and immature kernel (10 DAP) (Fig. 5b). All nine *ZmACBP* transcripts were detected in all tissues, and the qRT-PCR data agreed with the microarray data in many, but not all, cases. For example, *ZmACBP1*, *ZmACBP2*, and *ZmACBP5* were more highly expressed in the leaf than in other organs or tissues (Fig. 5b). *ZmACBP3* was more highly expressed in the kernel, and had almost the same expression level in others organs (Fig. 5b). *ZmACBP4* was relatively highly expressed in root, and showed lower expression in leaf (Fig. 5b). *ZmACBP6* had the highest expression level in cob, while *ZmACBP7* and *ZmACBP8* both strongly expressed in stem (Fig. 5b). *ZmACBP9* was relatively highly expressed in silk and 10 DAP of kernel, followed in stem, and showed the lowest expression in leaf (Fig. 5b).

### Regional association mapping for *ZmACBP* genes

**Fig. 6:**
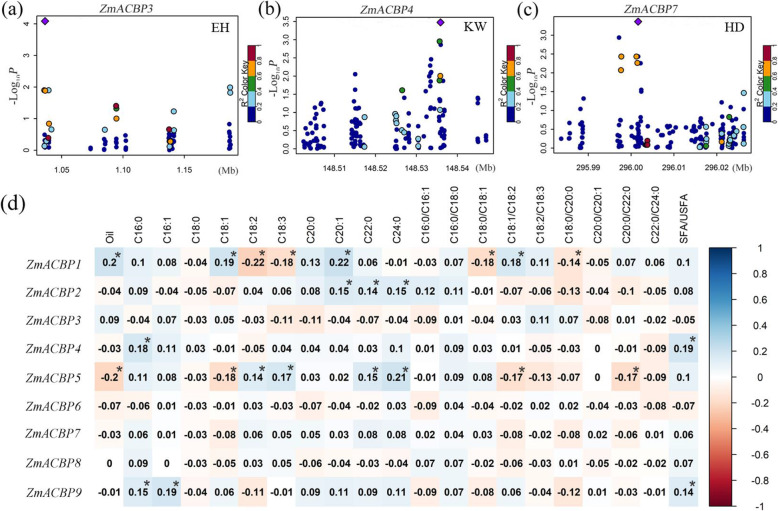
Regional associations of *ZmACBP* genes associated with agronomic traits. **a-c** Three *ZmACBP* genes, *ZmACBP3/4/7*, were significantly associated with ear height (EH), kernel width (KW), and heading date (HD), respectively. The circles of different colors represent different SNPs in each plot, and the purple diamond represents the most significantly associated SNP. The *x* axis indicates the SNP location, and the *y* axis shows the log10(*p*) (*P*-value). **d** Heat map showing the correlation among *ZmACBP* genes and the oil content in maize kernel. Blue and red colors on the heat map indicate positive and negative correlations, respectively. Asterisk indicates a significant correlation at ≤0.01 level between gene expression and oil accumulation

To explore the potential impact of the *ZmACBP* genes on agronomic traits in maize, an association mapping panel with 500 different maize inbred lines was used to determine whether there were any associations between *ZmACBP* genes and agronomic traits. A total of eight *ZmACBP* genes were found to be significantly correlated with one or more agronomic traits (*P* < 0.05), including plant height, ear height, ear diameter, 100 grain weight, silking time, heading date, kernel width, and others (Additional file 5: Table S3). Additionally, some *ZmACBP* genes were found to be strongly associated (*P* < 0.01) with important agronomic traits, such as grain yield, flowering time, and kernel-related traits. For example, *ZmACBP3* (Fig. 6a), *ZmACBP4* (Fig. 6b), and *ZmACBP7* (Fig. 6c) were significantly associated with ear height, kernel width, and heading date at *P* < 0.01, respectively. Furthermore, the correlation between *ZmACBP* expression levels and oil content in maize kernels was also investigated (Fig. 6d). At *P* ≤ 0.01, the expression levels of five genes (*ZmACBP1*, *ZmACBP2*, *ZmACBP4*, *ZmACBP5*, and *ZmACBP9*) were directly correlated with oil content in maize kernels (Fig. 6d; Additional file 6: Table S4). These results suggested that these *ZmACBP* genes could be related to maize development and kernel-related traits.

### Expression profiling of *ZmACBP* genes in response to various abiotic and biotic stresses

**Fig. 7:**
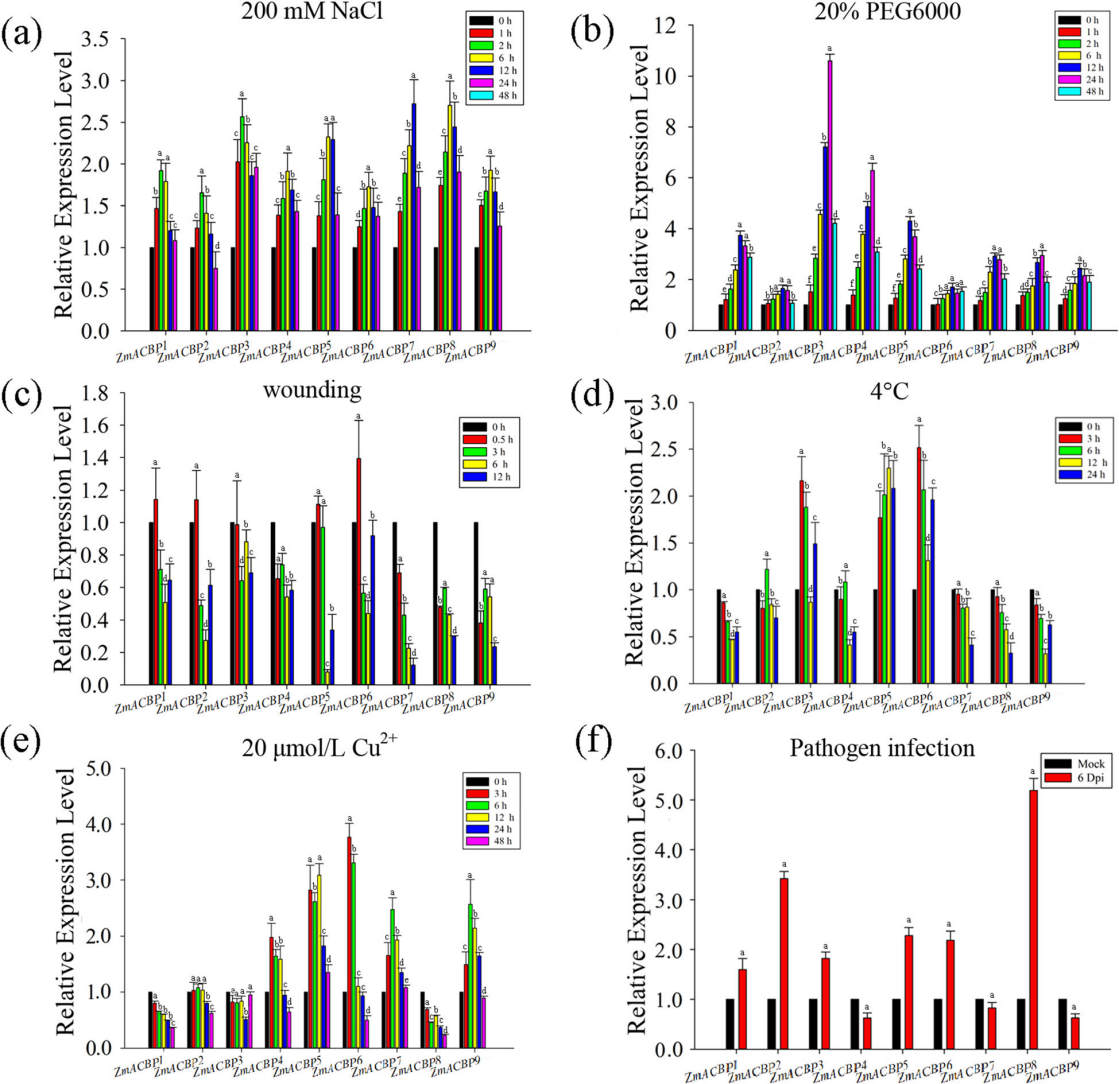
Quantitative real-time PCR analysis of *ZmACBPs* expression under stress treatments. **a** 200 mM NaCl, **b** 20% PEG6000, **c** wounding, **d** 4 °C, **e** 20 μmol/L Cu^2+^, and (**f**) infection with fungus infection (*Ustilago maydis*). The expression levels were normalized to that of TUB-ribosylation factor. Data represent mean ± SE of three replicates. Different letters indicate differences of the treatment compared to control at *P* < 0.01

To gain further insight into the role that *ZmACBP* genes may play in response to abiotic or biotic stresses, their expression patterns were assessed via qRT-PCR under different stress treatments, including high salinity (200 mM NaCl), osmotic stress (20% PEG6000, simulating drought), wounding, cold stress (4 °C), heavy metal ion stress (20 μmol/L Cu^2+^), and fungal infection (*Ustilago maydis*) (Fig. 7). The expression analysis revealed that several *ZmACBP* genes were induced by high salinity or osmotic stress treatments, peaking within 24 h after the treatments and then remaining at relatively high levels after the stress treatment (Fig. 7a and b). *ZmACBP1*, *ZmACBP2*, *ZmACBP5*, and *ZmACBP6* were all rapidly induced and peaked at 0.5 h after wounding treatment, then decreased to lower levels than control over the course of the treatment. The expression of *ZmACBP3, ZmACBP4, ZmACBP7, ZmACBP8*, and *ZmACBP9* was reduced during wounding treatment (Fig. 7c). The expression levels of *ZmACBP1*, *ZmACBP4*, *ZmACBP7*, *ZmACBP8*, and *ZmACBP9* decreased after cold treatment and then recovered somewhat at either 12 or 24 h (Fig. 7d). While *ZmACBP2*, *ZmACBP3*, *ZmACBP5* and *ZmACBP6* mRNAs were induced during cold treatment, reaching peak levels within 12 h, then remained at relatively high levels at 24 h (Fig. 7d). During copper ion treatment, *ZmACBP1* and *ZmACBP8* mRNAs were reduced within 48 h, and the level of *ZmACBP3* mRNA was mostly unaffected (Fig. 7e). *ZmACBP4*, *ZmACBP5*, *ZmACBP6*, *ZmACBP7*, and *ZmACBP9* were rapidly induced by copper ion treatment, with expression peaking at 3 or 6 h after copper ion treatment, then remaining at relatively high levels at 48 h (Fig. 7e). Fungal infection (*U. maydis*) caused an increase in the expression level of *ZmACBP1*, *ZmACBP2*, *ZmACBP3*, *ZmACBP5*, *ZmACBP6*, and *ZmACBP8*, while the expression levels of *ZmACBP4*, *ZmACBP7*, and *ZmACBP9* were reduced (Fig. 7f). These results indicate that ZmACBPs maybe play important roles in abiotic stress tolerance and biotic defense responses.

### The overexpression of two *ZmACBP* genes in *A. thaliana* enhanced the tolerance to stresses

**Fig. 8:**
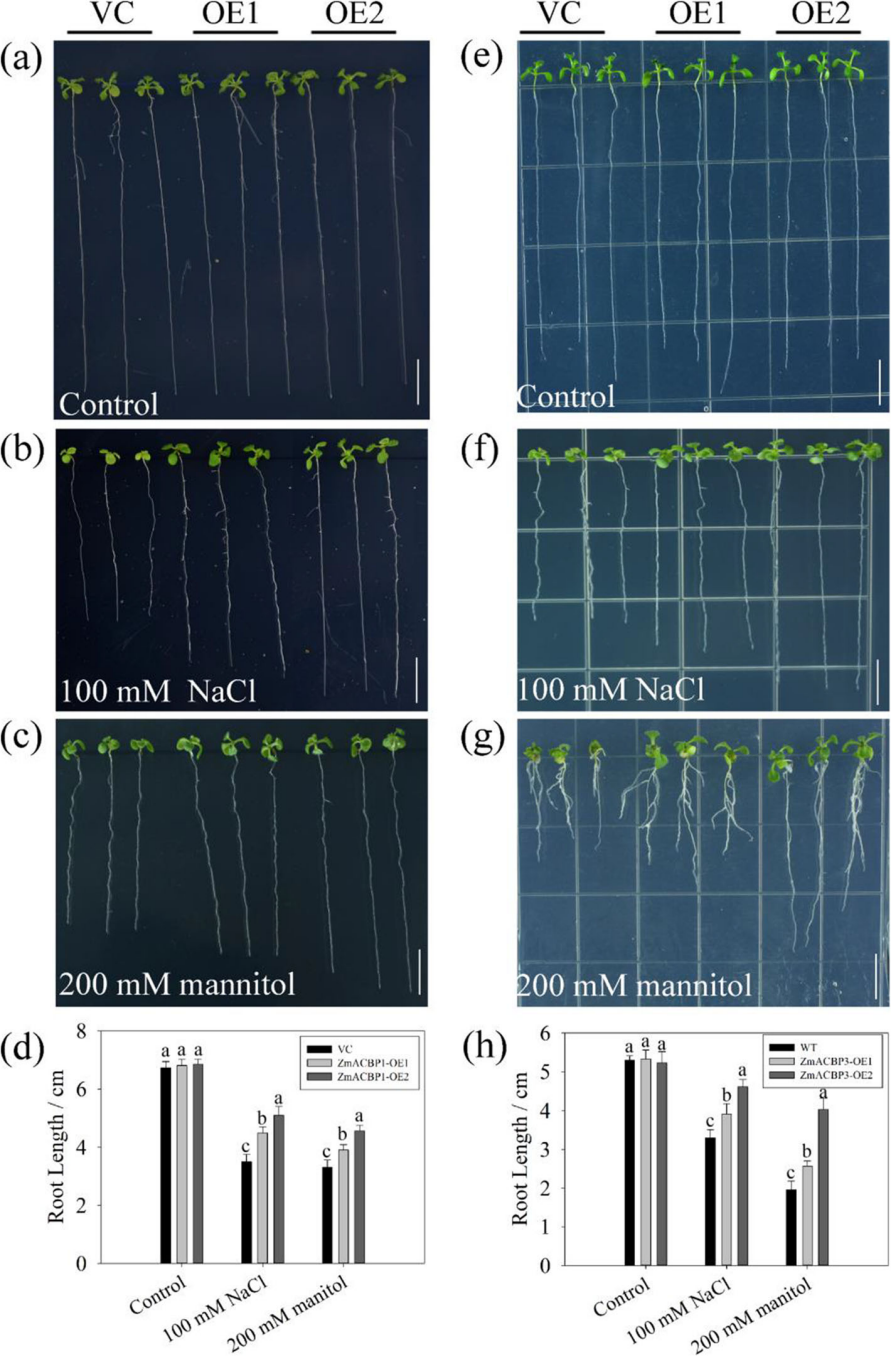
*ZmACBP1* and *ZmACBP3* positively regulated plant tolerance in *A. thaliana.* Phenotypes of 10-day-old vector control (VC) and *ZmACBP1* transgenic line (OE1 and OE2) seedlings under without stress (**a**), 100 mM NaCl (**b**), and 200 mM mannitol (**c**). **d** Root lengths of VC and *ZmACBP1* transgenic plants. Phenotypes of 10-day-old VC and *ZmACBP3* transgenic line (OE1 and OE2) seedlings under without stress (**e**), 100 mM NaCl (**f**), and 200 mM mannitol (**g**). **h** Root lengths of VC and *ZmACBP3* transgenic plants. Data represent mean ± SE of three replicates. Different letters indicate differences of transgenic lines compared to VC at *P* < 0.01

To confirm *ZmACBPs* response to stress, *ZmACBP1* and *ZmACBP3* were selected for further overexpression (OE) in *A. thaliana*. Since the expression levels of both *ZmACBP1* and *ZmACBP3* were induced by salinity and osmotic stress (Fig. 7a and b), the OEs were challenged with 100 mM NaCl or 200 mM mannitol (simulating drought). When no stress, there was no observable phenotypic difference between the OE lines and the vector control (VC) plants with respect to either leaf or root growth (Fig. 8a and e). However, when exposed to 100 mM NaCl or 200 mM mannitol, the OE lines overexpressing *ZmACBP1* or *ZmACBP3* grew more vigorously and had longer roots than the VC under (Fig. 8b-d; f-h).

### *ZmACBP3* overexpression altered the expression patterns of lipid metabolic genes and stress-responsive genes in *Arabidopsis*

**Fig. 9:**
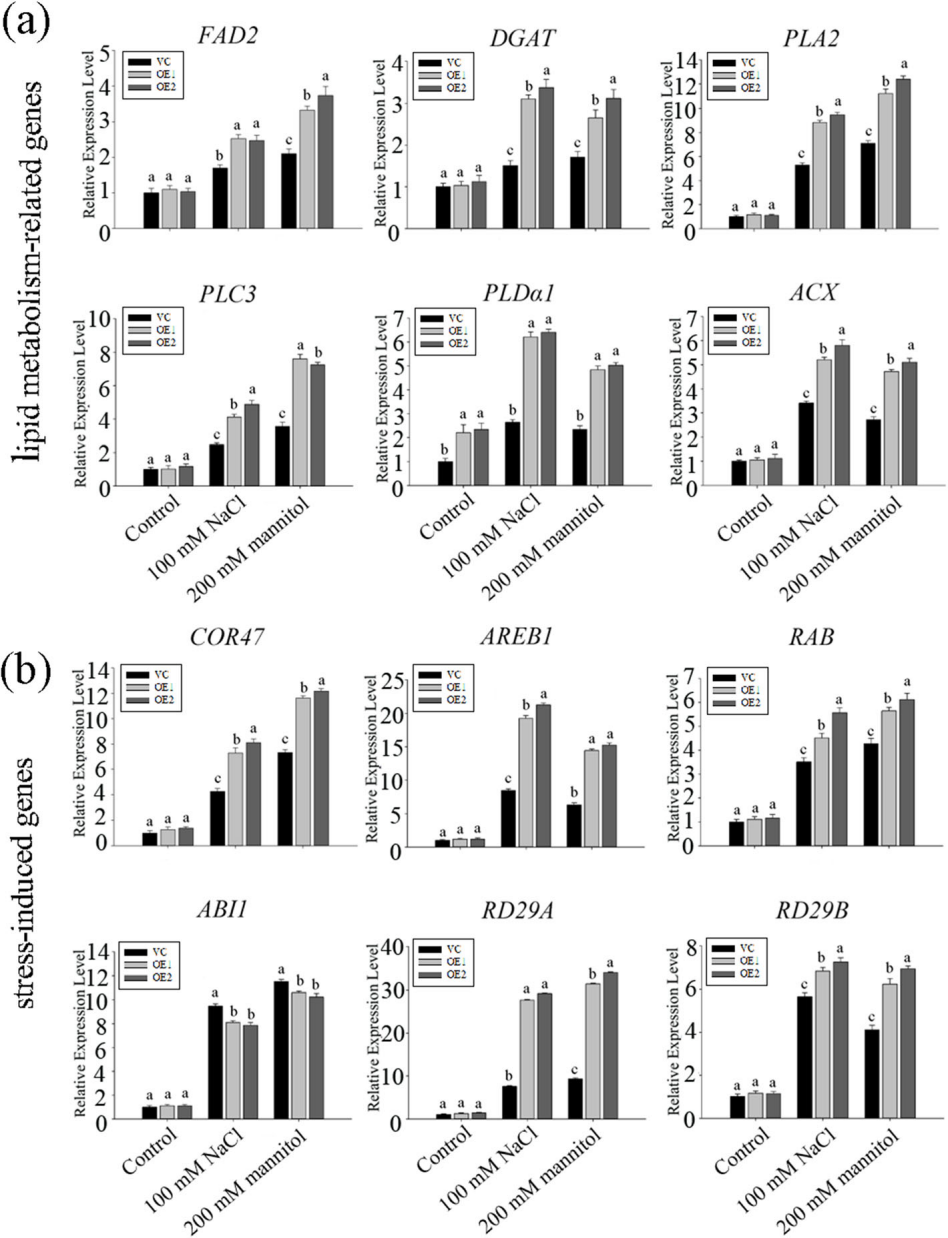
Expression patterns of lipid metabolism-related genes (**a**) and stress-responsive genes (**b**) in vector control (VC) and *ZmACBP3* transgenic *Arabidopsis* seedlings in response to 100 mM NaCl or 200 mM mannitol. Data represent mean ± SE of three replicates. Different letters indicate differences of transgenic lines compared to VC at *P* < 0.01

In order to explore the mechanism of *ZmACBPs* overexpression enhancing the tolerance to stress, the expression of lipid metabolic genes and stress-responsive genes were examined by qRT-PCR. Under normal conditions, the expression levels of the five lipid metabolic genes (*FAD2*, *DGAT*, *PLA2*, *PLC3*, and *ACX*) and all stress-responsive genes (*COR47*, *AREB1*, *RAB*, *ABI1*, *RD29A*, and *RD29B*) were similar in both the transgenic and wild type (WT) plants (Fig. 9a and b). The expression levels of the lipid metabolic or stress-responsive genes in both the *ZmACBP3* OE lines and the WT plants were significantly increased under salt or osmotic stress. However, compared with wild-type plants, the expression levels of these genes increased more significantly in the *ZmACBP3* OE line under 100 mM NaCl or 200 mM mannitol. (Fig. 9a and b). These results suggest that the *ZmACBP3* overexpression may alter lipid metabolism, which may act as signal molecules to increase the expression of stress-responsive genes, and ultimately leading to enhance the tolerance to stress in the *ZmACBP3* OE lines.

## Discussion

### Evolutionary conservation and variation of *ACBP* genes among different species

ACBPs have been functionally annotated in several higher plants, including Arabidopsis, rice, and *B. napus* [24]. In this study, *ACBP* genes were found to exist in all plants, including the green microalga, *Physcomitrella* moss, and other land plants (Fig. 1), indicating that these genes might originate in the green microalga and were relative conserved throughout the evolution of higher plants. However, the types and numbers of *ACBP* genes from different plant species were different (Fig. 1), which may be related to the functional specificity of various ACBPs in different plants. Nine ZmACBPs were identified in this study, which were then clustered into four different subfamily groups based on phylogenetic analysis (Additional file 1: Fig. S1; Fig. 2). Sequence alignment analysis shown that ZmACBP had a similar functional domains compared to with ACBPs homologues from *A. thaliana* (Additional file 1: Fig. S1a). Gene structure analysis of *ACBP* genes revealed that genes within the same branch generally possessed similar exon-intron structures (Fig. 2). In addition, the motif analysis of ACBPs also found that the ACBPs that were clustered in the same subfamily shared a similar motif composition (Fig. 2). The high degree of sequence similarity found across species implies that these genes likely share conserved functions.

### Expression patterns of *ZmACBP* gene family in maize under different tissues or stresses

ACBPs have been proposed to play several different roles in lipid trafficking, depending on their subcellular localization. In *A. thaliana* or rice, ACBPs were found to localize to different subcellular compartments, including the cytosol (AtACBP4/5/6 and OsACBP1/2/3) and the ER (AtACBP1/2 and OsACBP4/5) [13, 17]. In this study, ZmACBP1/5/7 were found to localize to the cytosol or plasma membrane (Fig. 4), which they may be involved in cytosolic acyl-CoA transport and maintenance of acyl-CoA esters during fatty acid biosynthesis. Given that the ER is an important place for the synthesis and transportation of phospholipids, ZmACBP3, which was localized to the ER (Fig. 7), may facilitate these processes. By binding acyl-CoA esters or phospholipids, ACBPs could modulate lipid metabolism and involve in various biological activities, including development and response to biotic or abiotic stresses [6]. For example, Arabidopsis AtACBP6 functions in maintaining the cytosolic acyl-CoA pool during seed and seedling development; AtACBP1 and AtACBP2 play an important role in protecting phospholipid membrane from peroxidation caused by heavy metal and oxidative stress [18]. The diverse localization patterns of ZmACBPs imply that they could be involved not only in the regulation of the growth and development of maize, but also various stress responses.

Consistent with studies of Arabidopsis and rice *ACBP* genes [13, 17, 18, 20], *ZmACBPs* genes had temporal and spatial specificity in different tissues and developmental stages (Fig. 5). This finding suggests that ZmACBPs might have multiple biological functions involving the growth and development of maize. In previous research, many plant ACBPs were found to respond to both abiotic and biotic stresses [16–20]. Different *ZmACBP* genes had variable responses to stress conditions (Fig. 7), which may be related to the presence of *cis*-elements in their promoter regions. For example, the *cis*-element of ABRE could be bound by ABA-responsive transcription factors (AREB/ABF) and is responsive to the ABA signaling pathway, while DRE/CRT is recognized by the C-repeat binding factor (CBF) family and plays an important role in responding to drought or salinity stress [25]. In this study, most of *ZmACBP* promoters that contained one or more ABRE or C-repeat/DRE (Fig. 3; Additional file 3: Table S2) were induced under salinity or osmotic stress (Fig. 7a and b), indicating that they may function in stress tolerance.

The variations in the expression of *ACBPs* could lead to changes in the abundance and composition of lipids under stress. For example, in Arabidopsis *AtACBP6*-overexpressors, the phospholipase Dδ (*PLDδ*) expression was increased under freezing stress [26]. PLDδ catalyzes the hydrolysis of phospholipids to phosphatidic acid (PA), while PA is involved in the osmotic stress, salt stress, and responses to ABA treatment as well as pathogen attack [27]. In addition, AtACBP interactors are reported to involve in plant development and stress response. AtRAP2.3 has been shown to interact with Arabidopsis Class II AtACBP2, which plays an important role in anoxia, oxidative (H_2_O_2_), osmotic (mannitol) and ABA-mediated stress responses [28]. In this study, the overexpression of *ZmACBP1* and *ZmACBP3* in *A. thaliana* enhanced the tolerance to salinity and osmotic stress (Fig. 8). Overexpression of *ZmACBP3* also increased the expression of *FAD2*, *DGAT*, *PLA2*, and others, which are involved in lipid metabolism (Fig. 9a). Furthermore, ectopic expression of *ZmACBP3* also dramatically increased the expression of several genes in stress signaling and response, including *COR47*, *AREB1*, *RAB*, and others (Fig. 9b). These genes play an important role in protecting plants from oxidative stress or osmotic stress [29]. Based on these results, it seems likely that *ZmACBP* overexpression may cause changes in the composition and metabolism of lipids, which in turn triggers lipid-dependent signaling cascades, ultimately changing the expression of stress-related genes and enhancing the tolerance to stresses.

### *ZmACBPs* are associated with important agronomic traits, with implications for maize breeding

In plants, ACBPs are involved in many stages of growth and development, including flowering, embryo development, seed germination and seedling development [24]. In previous work, Arabidopsis overexpressing grapevine *VvACBP1* was shown to have slower floral transition due to suppression of the photoperiodic pathway [30]. During embryogenesis in Arabidopsis, *AtACBP1* and *AtACBP2* have been shown to be involved in the synthesis of phospholipids and galactolipids in the ER [31]. Additionally, the overexpression of *B. napus ACBP1* affected the composition of fatty acids in Arabidopsis seeds, while *acbp4/acbp5/acbp6* mutants of Arabidopsis accumulated 18:1-CoA in the embryos and had decreased total seed weight [31]. Work in rice has shown that *OsACBP2*-OEs performed better than the WT and vector-transformed controls, with higher germination, seedling growth, grain size, and grain weight. Additionally, these *OsACBP2*-OE seeds had higher levels of triacylglycerols and LCFAs compared to control seeds [32]. Therefore, normal expression of ACBP genes is important to maintain normal morphology and kernel development in plants. In this study, a majority of *ZmACBP* genes were found to be constitutively expressed in different maize tissues (Fig. 5). Through correlation analysis, several *ZmACBP* genes were found to be associated with agronomic traits (Fig. 6; Additional file 5: Table S3), and the expression levels of multiple *ZmACBP* genes were correlated with oil content in maize kernel (Fig. 6; Additional file 6: Table S4). These findings have implications for genetic engineering of maize, raising the possibility of manipulating *ACBP* gene expression to improve yield or other related agronomic traits.

## Conclusions

In this study, nine members of the *Z. mays* ACBP family were identified. Various analyses of ZmACBPs, including evolution, gene structure, conserved motif, and subcellular localization, indicated that ZmACBPs were highly conserved across different species, and also had its own unique characteristics. qRT-PCR analyses revealed that *ZmACBPs* had tissue and organ-specific expression patterns and were also affected by various abiotic and biotic stresses. In addition, association analysis between *ZmACBP* genes and agronomic traits showed that some *ZmACBP* genes were significantly correlated with developmental and kernel-related traits. Overall, this study provides many new clues for further exploring the functions and breeding applications of the *Z. mays* ACBP family.

## Methods

### Plant materials and stress treatments

Maize inbred line W22 (*Zea mays* L, 2n = 20) was kindly provided by Dr. Jianbing Yan, Huazhong Agricultural University, China. The germination and growth of maize seeds were carried out as described previously [33]. In brief, maize seeds were first surface sterilized with 75% alcohol for 5 min and washed with distilled water three times, then incubated on moistened filter paper at 28 °C for germination. The seedlings were cultivated to the three-leaf stage with Hoagland’s solution in 28 °C greenhouse with a regime of 16 h light and 8 h dark, and then selected for stress treatment. For the osmotic treatment, the seedlings were treated with 20% PEG 6000 and collected at 0, 3, 6, 12, 24, and 48 h [33]. For the salinity treatment, the seedlings were subjected to 200 mM NaCl and harvested at 0, 3, 6, 12, 24, and 48 h [33]. For the cold treatments, seedlings at the three leaves stage were incubated at 4 °C for 0, 3, 6, 12, and 24 h [34]. For the heavy metal treatments, the seedlings were subjected to 20 μmol/L Cu^2+^ copper solutions and harvested at 0, 3, 6, 12, 24, and 48 h. For wounding treatment, leaves of seedlings were scratched using a needle. Wounded leaves were subsequently maintained on water-saturated filter paper and sampled at 0, 0.5, 2 and 24 h after treatment [35]. In fungus infection (*Ustilago maydis*), pathogenicity assays were performed as described [36], and maize leaves were sampled at 6 day post infection (dpi). Each treatment consisted of three replicates. Adult plants were grown in the field, and then all organs and tissues were harvested. The root was harvested at the three leaves stage; the stem and leaf were collected at five fully extended leaves; the silk, cob and anther were harvested at 13 extended leaves; the kernel was harvested at 10 DAP. All materials were frozen in liquid nitrogen immediately after collected and stored − 80 °C for RNA extraction.

### Identification and phylogenetic analysis of the *ACBP* genes in maize

The Hidden Markov Model (HMM) profile with the ACBP domain (PF00887) from PFam (http://pfam.sanger.ac.uk/) was used as query sequences in local HMM-based searches to identify putative ZmACBP proteins sequences, with a cut-off expected value (E-value) of 10^− 5^. In order to avoid the ZmACBP proteins that might be missed by the HMM model searching, the *A. thaliana* or rice ACBP protein sequences were used to further search ZmACBP proteins from the Maize Genome Database (https://www.maizegdb.org), Phytozomev12.0 (http://www.phytozome.net), and EMBL (https://www.ebi.ac.uk/). The matched ZmACBP protein sequences were further confirmed and analyzed using SMART (http://smart.embl.de/). To study the phylogenetic relationships of ACBP in different plant species, ACBP sequences from maize, Arabidopsis, rice, *Triticum aestivum*, *Brachypodium distachyon* and *Glycine max* were used to construct a phylogenetic tree using MEGA7.1 by the Neighbor Joining (NJ) method with 1000 bootstrap replicates. All sequences were listed in Additional file 7: Data Set S1.

### Chromosomal distribution, gene structure and protein characteristics of the *ACBP* genes in maize

The chromosomal distribution was mapped by MapGene2Chromosome web 2.0 (http://mg2c.iask.in/mg2c_v2.0/). A synteny analysis map within the maize and rice genomes were identified using the TBtools [37]. The Gene Structure Display Server v2.0 (http://gsds.cbi.pku.edu.cn/) was used to analyze the gene structure and generate the exon/intron organization. The Plant CARE database (http://bioinformatics.psb.ugent.be/webtools/plantcare/html/) were used to predict and analyze the *cis*-regulatory elements of 1500 bp upstream sequences of each maize *ACBP* genes [38]. The compute pI/Mw tool (http://web.expasy.org/compute_pi/) was used to evaluate the protein pI/Mw. The online Multiple Em for Motif Elicitation (MEME, http://meme.sdsc.edu/meme4_3_0/intro.html) was used to identify the conserved motifs.

### Subcellular localization of ZmACBPs

The subcellular locations of selected ZmACBPs were determined according to the Wang et al. 2018 [39]. The coding sequences of *ZmACBPs* genes were performed to amplify using gene-specific primers (Additional file 8: Table S5), then fused in-frame to the C terminus of GFP (green fluorescent protein) vector using the Gateway LR reaction (Invitrogen). The conjugated vectors were transformed into *Agrobacterium tumiefacien*s strain GV3101, then the bacteria harboring relevant plasmids were infiltrated into *N. benthamiana* leaves following Liu et al. [40]. Additionally, ZmACBPs minus the stop codons were cloned and inserted into pBI221:eGFP, and the corresponding expression vectors were introduced into Arabidopsis protoplasts according to Zhang and Wu [41], with minor revision. The confocal laser scanning microscope LSM 800 (Zeiss) was used to detect the fluorescent signal of ZmACBP-GFP fusion proteins.

### Expression analysis and quantitative real-time PCR

The RNA-seq data of the *ZmACBP* genes was retrieved from the MaizeGDB platform (http://bar.utoronto.ca/efp_maize/cgi-bin/efpWeb.cgi?dataSource=Sekhon_et_al_Atlas) with the Maize Gene Chip platform. A complete linkage hierarchical cluster analysis based on the Pearson coefficients with average linkage was carried out by Heatmapper (http://www.heatmapper.ca/).

Quantitative real-time PCR was carried out as described by Lin et al. [33], with minor revision. According to the user manual, the Trizol reagent (Takara, Dalian, China) was used to extract total RNAs from different maize tissues (root, silk, stem, leaf, cob, anther, and kernel), and the RNA from each tissue was reverse-transcribed into cDNA using the PrimerScript RT reagent kit with a gDNA eraser (Takara, Dalian, China). The specific primers of all analyzed genes were designed by Primer Premier 5.0 and listed in Additional file 8: Table S6, and TUB-ribosylation factor was selected as the internal reference for normalization. The qRT-PCR reaction was performed on Bio-Rad CFX Connect™ using SYBR-Green, and the expression abundances of target genes were calculated by the 2^(−ΔΔCt)^ analysis method [33]. All reactions were performed in triplicate.

### Regional association mapping for *ZmACBP* genes

Regional association tests were performed in an association mapping panel composed of 500 inbred lines [42]. The genotype of this panel was detected by two genotyping platforms, resulting in 550,000 high quality SNPs [43, 44]. The 17 agronomic traits included plant height, ear height, tassel branch number, ear diameter, 100 grain weight, silking time, heading date, leaf number above ear, tassel main axis length, ear length, kernel width, cob weight, pollen shed, kernel weight, kerner number perrow, ear leaf width, ear leaf length.

Only SNPs within the range of 100 kb upstream and downstream of candidate genes were used. And then an association mapping analysis was conducted using a mixed liner model that took into account population structure and relative kinship to test for statistical association between agronomic traits phenotypes and genotypes of each *ZmACBP* gene. Association mapping analysis and linkage disequilibrium (LD) among SNPs were conducted in TASSEL (v 5.0) [45]. The physical location of the SNPs was identified based on the maize genomic sequence version 5b.60 [46]. The correlation among *ZmACBP* genes expression levels and the oil content in maize kernel (Pearson’s R correlations) was identified by using the linear model function in R package ‘stats’ (version 2.15.3), which data were retrieved from Fu et al. 2013 [42] and Li et al. 2013 [44]. The *p*-values were adjusted for multiple comparisons using the method of Klipper-Aurbach [47]. The cut-off values for significantly expressed genes were the adjusted p-values (*p* < 0.01). Heat maps of the genes were constructed by the OmniViz Treescape software.

### Transformation of *A. thaliana* and stress treatment of transgenic plants

To overexpress *ZmACBP1 or ZmACBP3* in Arabidopsis, the coding region of *ZmACBP1/3* were ligated into pSTART vector, and transformed into *A. thaliana* ecotype Col-0 via the *Agrobacterium tumefaciens*-mediated floral-dip method [48]. The growth conditions and stress treatments applied to Arabidopsis were carried out as described previously [49], with minor revision. Arabidopsis seeds from vector control (VC) lines and overexpression (OE) lines were germinated on the surface of half-strength Murashige and Skoog (1/2MS) agar medium. The plates were kept at 4 °C in the dark for 3 days to break seed dormancy, and then removed to a 16 h photoperiod and a 22 °C temperature for 2 days. The uniform seedlings were moved to fresh medium with either 100 mM NaCl or 200 mM mannitol for 10 days. All these experiments were performed in triplicate.

## Supplementary Information

**Additional file 1: Figure S1.** The domain structures, genome distribution and synteny analysis of *ZmACBP* genes. (a) The domain structures of the maize and Arabidopsis ACBPs. Boxes of different colors represent different domains: blue, the ACB domain; green, ankyrin repeats; pink, kelch motifs. (b) The chromosomal distributions of the nine *ZmACBP* genes. Mb: million base. The number of the chromosome is shown at the top of each chromosome. The name and the location of each *ZmACBP* gene were shown on the left and right side of the chromosome. The box indicates the tandem duplicated gene. (c) Synteny analysis of *ACBP* genes from maize and rice. Gray lines in the background indicate the collinear blocks within maize and rice genomes, while the red lines highlight the syntenic *ACBP* gene pairs.

**Additional file 2: Table S1.** Synteny blocks of *ACBP* genes between maize and rice genomes.

**Additional file 3: Table S2.** Functions and number of identified *cis*-regulatory elements of *ZmACBP* genes.

**Additional file 4: Figure S2.** The subcellular localization of selected ZmACBPs in Arabidopsis leaf protoplasts. pBI221-eGFP: empty vector. Scale bar: 20 μm.

**Additional file 5: Table S3.** The *ZmACBP* genes list associated with agronomic traits in maize.

**Additional file 6: Table S4.** The correlation among *ZmACBP* genes expression levels and oil content in maize kernel.

**Additional file 7: Data Set S1.** The protein sequences used to generate phylogenetic tree.

**Additional file 8: Table S5–6.** Primers and their sequences used in this study.

## Data Availability

Acquired sequences of ZmACBP proteins are available from EMBL/GenBank under accession no. GCA_902167145 and included in Additional files 7 of this article. The datasets analyzed for regional association or phenotype data during the current study are available in the Maize Group-Resources (http://www.maizego.org/Resources.html).

## Abbreviations

ACBP: Acyl-CoA-binding proteins
GFP: Green fluorescent protein
MW: Molecular weight
PEG: Polyethylene glycol
qRT-PCR: Real-time quantitative reverse transcriptional-polymerase chain reaction.
