# Supplementary figures and images for: Molecular characterization, expression and functional analysis of acyl-CoA-binding protein gene family in maize (Zea mays)
Source: BMC Plant Biol. 2021 Feb 15;21:94. doi: 10.1186/s12870-021-02863-4 (PMC7883581; doi:10.1186/s12870-021-02863-4)

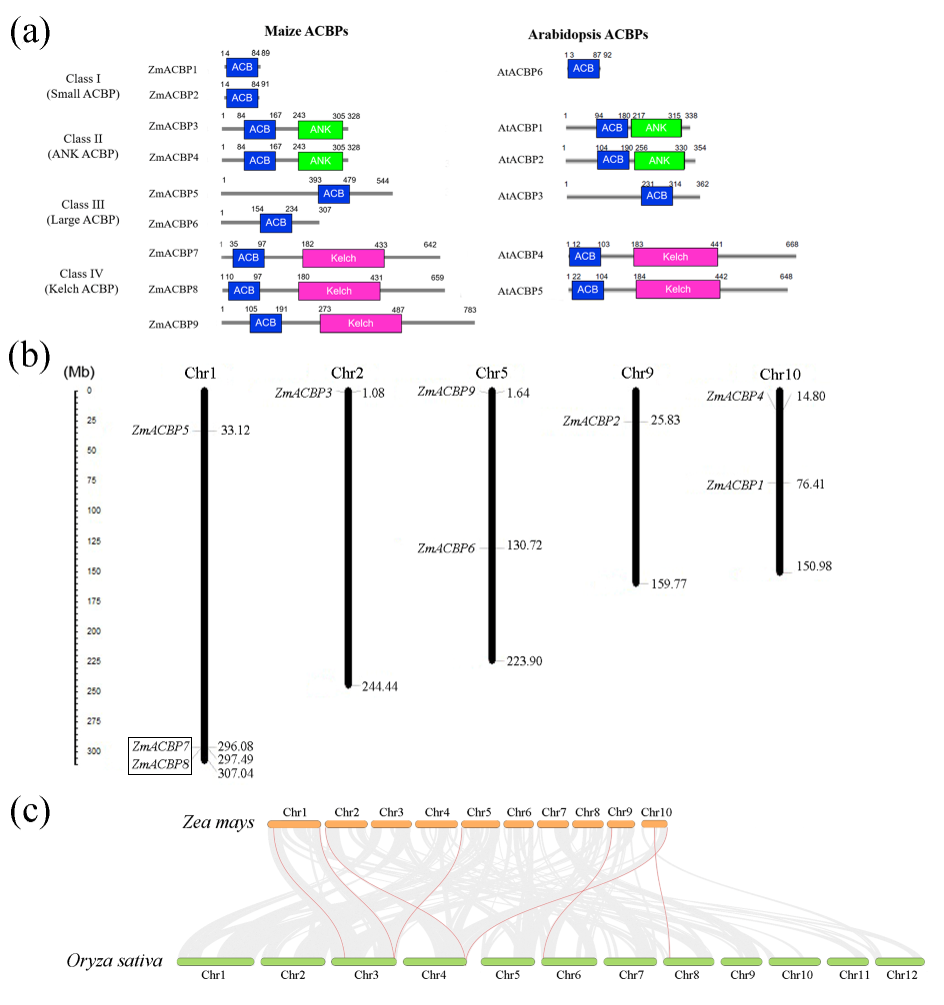

Supplement: Supplementary file 1 — Additional file 1: Figure S1. The domain structures, genome distribution and synteny analysis of ZmACBP genes. (a) The domain structures of the maize and Arabidopsis ACBPs. Boxes of different colors represent different domains: blue, the ACB domain; green, ankyrin repeats; pink, kelch motifs. (b) The chromosomal distributions of the nine ZmACBP genes. Mb: million base. The number of the chromosome is shown at the top of each chromosome. The name and the location of each ZmACBP gene were shown on the left and right side of the chromosome. The box indicates the tandem duplicated gene. (c) Synteny analysis of ACBP genes from maize and rice. Gray lines in the background indicate the collinear blocks within maize and rice genomes, while the red lines highlight the syntenic ACBP gene pairs. [file 12870_2021_2863_MOESM1_ESM.tif]

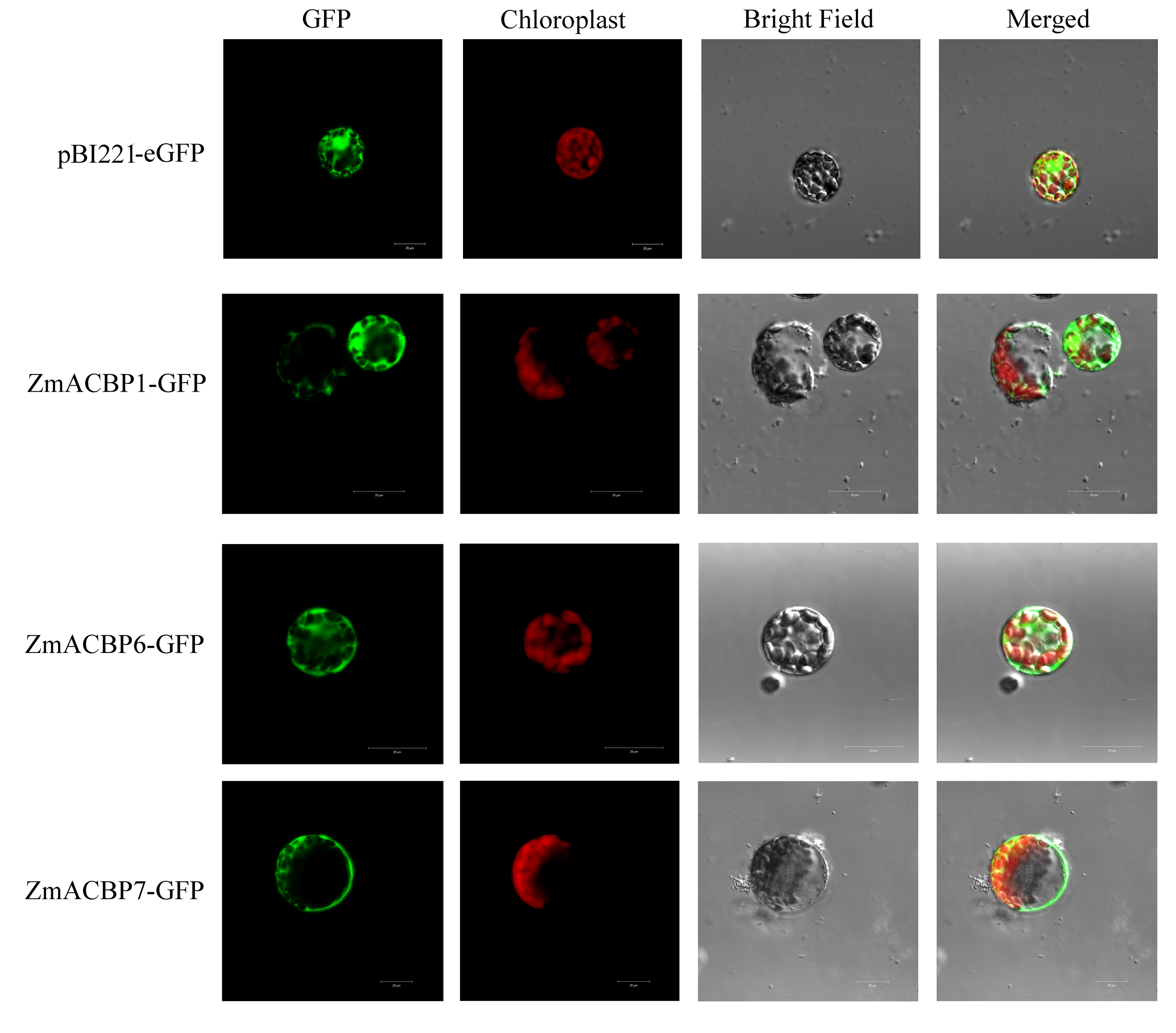

Supplement: Supplementary file 4 — Additional file 4: Figure S2. The subcellular localization of selected ZmACBPs in Arabidopsis leaf protoplasts. pBI221-eGFP: empty vector. Scale bar: 20 μm. [file 12870_2021_2863_MOESM4_ESM.tif]
