# Supplementary material for: Molecular characterization, expression and functional analysis of acyl-CoA-binding protein gene family in maize (Zea mays)
Source: BMC Plant Biol. 2021 Feb 15;21:94. doi: 10.1186/s12870-021-02863-4 (PMC7883581; doi:10.1186/s12870-021-02863-4)
Supplement: Supplementary file 7 — Additional file 7: Data Set S1. The protein sequences used to generate phylogenetic tree. [file 12870_2021_2863_MOESM7_ESM.docx]

> At4g27780 (AtACBP1)

MGDWAQLAQSVILGLIFSYLLAKLISIVVTFKEDNLSLTRHPEESQLEIKPEGVDSRRLD

SSCGGFGGEADSLVAEQGSSRSDSVAGDDSEEDDDWEGVESTELDEAFSAATLFVTTAAA

DRLSQKVPSDVQQQLYGLYKIATEGPCTAPQPSALKMTARAKWQAWQKLGAMPPEEAMEKYIEIVTQLYPTWLDGGVKAGSRGGDDAASNSRGTMGPVFSSLVYDEESENELKIDAIHGFAREGEVENLLKSIESGIPVNARDSEGRTPLHWAIDRGHLNIAKVLVDKNADVNAKDNEGQTPLHYAVVCDREAIAEFLVKQNANTAAKDEDGNSPLDLCESDWPWIRDSAKQAD

> At5g53470 (AtACBP2)

MADWYQLAQSIIFGLIFAYLLAKLISILLAFKDENLSLTRNHTTQSEYENLRKVETLTGI

SGETDSLIAEQGSLRGDEDESDDDDWEGVESTELDEAFSAATAFVAAAASDRLSQKVSNE

LQLQLYGLYKIATEGPCTAPQPSALKMTARAKWQAWQKLGAMPPEEAMEKYIDLVTQLYPAWVEGGSKRRNRSGEAAGPMGPVFSSLVYEEESDNELKIDAIHAFAREGEVENLLKCIEN

GIPVNARDSEGRTPLHWAIDRGHLNVAEALVDKNADVNAKDNEGQTSLHYAVVCEREALAEFLVKQKADTTIKDEDGNSPLDLCESEWSWMREKKDSN

> At4g24230 (AtACBP3)

MEVFLEMLLTAVVALLFSFLLAKLVSVATVENDLSSDQPLKPEIGVGVTEDVRFGMKMDA

RVLESQRNFQVVDENVELVDRFLSEEADRVYEVDEAVTGNAKICGDREAESSAAASSENY

VIAEEVILVRGQDEQSDSAEAESISSVSPENVVAEEIKSQGQEEVTELGRSGCVENEESG

GDVLVAESEEVRVEKSSNMVEESDAEAENEEKTELTIEEDDDWEGIERSELEKAFAAAVN

LLEESGKAEEIGAEAKMELFGLHKIATEGSCREAQPMAVMISARAKWNAWQKLGNMSQEEAMEQYLALVSKEIPGLTKAGHTVGKMSEMETSVGLPPNSGSLEDPTNLVTTGVDESSKNEIVSGER

> At3g05420 (AtACBP4)

MAMPRATSGPAYPERFYAAASYVGLDGSDSSAKNVISKFPDDTALLLYALYQQATVGPCN

TPKPSAWRPVEQSKWKSWQGLGTMPSIEAMRLFVKILEEDDPGWYSRASNDIPDPVVDVQINRAKDEPVVENGSTFSETKTISTENGRLAETQDKDVVSEDSNTVSVYNQWTAPQTSGQRPKARYEHGAAVIQDKMYIYGGNHNGRYLGDLHVLDLKSWTWSRVETKVATESQETSTPTLLAPCAGHSLIAWDNKLLSIGGHTKDPSESMQVKVFDPHTITWSMLKTYGKPPVSRGGQSVTMVGKTLVIFGGQDAKRSLLNDLHILDLDTMTWDEIDAVGVSPSPRSDHAAAVHAERFLLIFGGGSHATCFDDLHVLDLQTMEWSRPAQQGDAPTPRAGHAGVTIGENWFIVGGGDNKSGASESVVLNMSTLAWSVVASVQGRVPLASEGLSLVVSSYNGEDVLVAFGGYNGRYNNEINLLKPSHKSTLQTKTLEAPLPGSLSAVNNATTRDIESEVEVSQEGRVREIVMDNVNPGSKVEGNSERIIATIKSEKEELEASLNKERMQTLQLRQELGEAELRNTDLYKELQSVRGQLAAEQSRCFKLEVDVAELRQKLQTLETLQKELELLQRQKAASEQAAMNAKRQGSGGVWGWLAGSPQEKDDDSP

> At5g27630 (AtACBP5)

MAHMVRASSGLSYPERFYAAASYVGLDGSQSSVKQLSSKFSNDTSLLLYTLHQQATLGPC

SIPKPSAWNPVEQSKWKSWQGLGTMPSIEAMRLFVKILEEADPGWYPRTSNSVLDPAVHV

QINSTKAEPSFESGASFGETKTITSEDGRLTETQDKDVVLEDPDTVSVYNQWTAPRTSGQ

PPKARYQHGAAVIQDKMYMYGGNHNGRYLGDLHVLDLKNWTWSRVETKVVTGSQETSSPAKLTHCAGHSLIPWDNQLLSIGGHTKDPSESMPVMVFDLHCCSWSILKTYGKPPISRGGQSVTLVGKSLVIFGGQDAKRSLLNDLHILDLDTMTWEEIDAVGSPPTPRSDHAAAVHAERYLLIFGGGSHATCFDDLHVLDLQTMEWSRHTQQGDAPTPRAGHAGVTIGENWYIVGGGDNKSGASKTVVLNMSTLAWSVVTSVQEHVPLASEGLSLVVSSYNGEDIVVAFGGYNGHYNNEVNVLKPSHKSSLKSKIMGASAVPDSFSAVNNATTRDIESEIKVEGKADRIITTLKSEKEEVEASLNKEKIQTLQLKEELAEIDTRNTELYKELQSVRNQLAAEQSRCFKLEVEVAELRQKLQTMETLQKELELLQRQRAVASEQAATMNAKRQSSGGVWGWLAGTPPPKT

> At1g31812 (AtACBP6)

MGLKEEFEEHAEKVNTLTELPSNEDLLILYGLYKQAKFGPVDTSRPGMFSMKERAKWDAWKAVEGKSSEEAMNDYITKVKQLLEVAASKAST

> Glyma04g14650 (GlACBP1)

MVMQEDFEQYAEKAKTLPPTQSNEDLLILYGLYKQATVGPVNTSRPGMFNMRDRAKWDAWKAVEGKSKDEAMSDYITKVKQLLEAAGMPA

> Glyma09g34770 (GlACBP2)

MDMQEDFEQYAEKAKTLPPTQSNEDLLILYGLYKQATVGPVNTSRPGMFNMRDRAKWDAWKAVEGKSKDEAMGDYIIKVKQLLEAAGLPA

> Glyma06g13630 (GlACBP3)

MAEWQSLLQSILVGLIFSYLLAKLISIVVSFKDDNLTVTRASAAETTATRAEQFEDALKR

DDAVSSDARPFEEESVVAEHGSVRIDSDGDYDDDWEGVESTELDEAFSAATAFVAAAAAD

RLSQKVSSDVQLQLYGLYKIATEGPCSTPQPSPLKMTARAKWQAWQKLGAMPPEDAMQKYIDIVTEIYPTWLDGSSLRNKSGDSGGHGSEAKGPMGPVFSTFVYEEEYGSDSQMEAIHGF

AREGDMANLLKCIENGVSMNLKDSEGRTPLHWAVDRGHLNVTELLVGKNADVNAKDNDGQTPLHYAVTCEREAIAEYLVKHNADIYSKDNDGSSPRDICESNWPCMQHVGEEVN

> Glyma04g41220 (GlACBP4)

MAEWQSLLQSIFVGLIFSYLLAKLISIVVSFKDDNLTVTRAAAAETTTTTRDDAVSSDAR

PFEEESMVAEHGSVRNDSDGDYDDDDWEGVESTELDEAFSAATAFVDAAAADRLSQKVSSDVQLQLYGLYKIATEGPCSTPQPSPLKMTARAKWQAWQKLGAMPPEDAMQKYIDIVTETYPTWLDGSSLRNKSGDSGGHGSEAKGPMGPVFSTFVYEEEYGSDSQMEAIHGFAREGDMANLLKCIENGVSMNLKDSEGRTPLHWAVDRGHLNVTELLVGKNADVNAKDNDGQTPLHYAVTCEREAIAEYLLKHNADIYSKDNDGSSPRDICESNWPCMQHVGGEVN

> Glyma17g35480 (GlACBP5)

MELLWELAFTIALSLLLPLVFLKLLSVTPNFEANEKVAVIRRDHDHRVESDSNSWETDKV

VQIGGKIDEFRDKPIVGKLVVPEIVDVSCGSPKIHNSEKIDGDRLHNEIELEDLAEDPVV

DEGNEGVVNINKVEVELMECDSRENKVEEVEISQCERYYNEIEESSMNEEMGENKGSVVDEDDWEGIERTELERRFGAAVVFVGSKSNANLSNDVKMKLHGYHRIATQGPCHEPQPMALKFSARAKWIAWRQLGIMSPEEAMEQYISLLSENIPDWIVENPYDNAKPASAKLTL

> Glyma14g09680 (GlACBP6)

MELLTVVDDFFVTASLALLLTFIVLKLVEVINDTHAIPKRHVVHREPDRPVSHAEQRFTV

QPAPAKTKVGFVSPVQEEFATCAVGTEHKIEKKAAVKPVRPRGKRFTVHPAQSKSGVETE

CNREEIAVEPERKIEEAVVEPVPVQTEVTVGLISPVQDDTCVGTAEKIEEATTVESDKNV

EEEIAEGNEGTQELDDSAEKRNVKSVEEISVEPSTEIEASVTDSGVKENFDDDDDDWEGI

ERSELEKEFMAATKFVSGEENRLGGAGSNLRMELYGLHKVATEGPCREPQPMALKLAARAKWNAWQKLGNMNPEVAMEQYVSLLSDKFPGWMKDTSAGIGEHETTRPEVSESAASDLSTTLSNQQQMITTERELEQESDSKDRSPLTVSDLENSVNK

> Glyma11g05360 (GlACBP7)

MELLWELAFTIALSLLLPLVFLKLLSVTPNLEANEKVALLGRDHDHGIKSDSKSWETDKV

VRIGGKIDEFRDKPIVGKLIVPEIVDVSCGSPKIHNSEKIDEYSVYDEIELVDLAEDPVV

DEGNDGVVNIDEVEVELMECDSRENKVEEVEISQCERNYNEIDESSMNEEIGENKGSVVD

EDDWEGIESTELERRFGAAVVFVGSKSNANLSNDVKMKLHGYHRIATQGPCHEPQPMALKFSARAKWIARRQLGIMSPEEAMEQFISLLSESIPDWIEENPYDNAKPASATNLVI

> Glyma01g39920 (GlACBP8)

MELLWELAFTIALSLLLPLVFLKLLSVTPNFEANEKVAVIRRDHDHRVESDSNSWETDKV

VQIGGKIDEFRDKPIVGKLVVPEIVDVSCGSPKIHNSEKIDGDRLHNEIELEDLAEDPVV

DEGNEGVVNINKVEVELMECDSRENKVEEVEISQCERYYNEIEESSMNEEMGENKGSVVDEDDWEGIERTELERRFGAAVVFVGSKSNANLSNDVKMKLHGYHRIATQGPCHEPQPMALKFSARAKWIAWRQLGIMSPEEAMEQYISLLSENIPDWIVENPYDNAKPASAKLTL

> Glyma20g37940 (GlACBP9)

MARASSGLQYPERFYAAASYVGFDGSTKSLTSKFSNSTALLLYSLYQQASIGPCNVPEPS

SWKLVEHSKWASWNQLGNMSSTEAMRLFVKILEEEDPGWYSRASNSVLDPVVDVQMNHNSKVEPVIENANAYPEIKTISTENGSHVGTQDKDVVIEGFGSVGVYDQWVAPPVSGQRPKARYEHGAAVVQDKLYIYGGNHNGRYLNDLHVLDLRSWTWSKIEAEVVESTNSSSITFPCAGHSLIPWENKLLSIAGHTKDPNESIQVKVFDLPNATWTTLKTYGKPPVSRGGQSVTFVGTSLVIFGGEDAKRTLLNDLHILDLETMTWDEIDAVGVPPSPRSDHAAAVHVERYLLIFGGGSH

ATCYNDLHVLDMQTMEWSRPTQLGEIPTPRAGHAGVTVGENWFIVGGGDNKSGVSETVVLNMATLTWSVVTSVQGRVPVASEGSSLVVSSYDGEDILVSFGGYNGHYNNEVYVLKPSHKSTLQSKLIENPIPYSVSGAHNAANATRDLDSEAGHKGIIKELVMDSVDSIKSKGDVITVLK

VEKEDLESSLYKEKLQTLQLKQELSETKTRNSDLCKELQSIRGQLASEQSRCFKLEVEVA

ELSQKLQTIGTLQKELELLQRQKAASELAALNAKQKQGSGGVWGWLAGAPPPTQKEDDG

> Glyma03g39640 (GlACBP10)

MAMARATSGLQYPERFYAAASYVGFDGSSPTKTLTSKFAKSTALLLYSLYQQASVGPCNI

PEPSTWKLVEHSKWASWNQLGNMSSTEAMRLFVKILEEEDPGWYSRASNSVVEPVIDVQMNQNSKVEPVIENGNSYPETKTISTQNGSEVGTQDKDIVVEGFGSVEVYDQWIAPPVSGGNPKARYEHGAAVVQDKLYIYGGNHNGRYLNDLHVLDLRSWTWSKIEAKTGVESPTTSIPCAGHSLIPWGNKLLSIAGHTKDPSESIQVKEFDLETAAWSTLKIFGKAPVSRGGQSVNLVGK

TLVIFGGQDAKRTLLNDLHILDLETMTWDEIDAVGVPPSPRSDHTAAVHVDRYLLIFGGG

SHATCYNDLHVLDLQTMEWSRPTQLGEIPSPRAGHAGVTVGENWFIVGGGDNKSGVSETVVLNMSTLTWSVVTSVQGRVPVASEGLSLVVSSYDGEDILVSFGGYNGRYNNEVYVLKPSHKSTLQSKIIENSIPDSVSAIPNVTNVESEFEAGHDADPPVCIIDADPPKSKGDLVSVLKA

EKEELESSLSKEKQHALQLKQDLVEAESRNSDLYKELQSVRGQLASEQSRCFKLEVEVAE

LGQKLQTIGTLQKELELLQRQKAASEQAALNAKQRQSSGGVWGWLAGTPPPSRNADDA

> Glyma19g42270 (GlACBP11)

MARASSGLQYPERFYAAASYVGFDGSSPTKSLTSKFPQSTALLLYSLYQQASVGPCNITE

PSTWKIVEHGKWASWNQLGNMSSTEAMRLFVKILEEEDPGWYSRLSNSVVEPVVDVQMNHNSKVEPVIENGNSYPETKTISISTQNGSEVGTQDKDTVVEGFGSVEVYDQWIAPPVSGGSPKARYEHGAAVVQDKLYIYGGNHNGRYLNDLHVLDLRSWTWSKIEAKTGVESPTTSIPCAGHSLIPWGNKLLSIAGHTKDPSESIQVKVFDLQMATWSTPKIFGKAPVSRGGQSVNLVGKTLVIFGGQDAKRTLLNDLHILDLETMTWDEIDAVGVPPSPRSDHTAAVHVERYLLIFGGG

SHATCYNDLHVLDLQTMEWSRPMQLGEIPTPRAGHAGVTVGENWFIVGGGDNKSGVSETIVLNMSTLAWSVVTSVQGRVPVASEGLSLVVSSYDGEDVLVSFGGYNGRYNNEVYVLKPSHKSTLQSKIIENSIPDSVSAIPNVTNVESEFEAGHDSNPPKSKGDIVSVLKAEKEELESSL

IKEKHHALQLKQDLAEAESCNSDLYKELQSVRGQLASEQSRCFKLEVEVAELGQKLQTIG

TLQKELELLQRQKAASEQAALNAKQRQSSGGVWGWLAGTPPPIQNADDA

> GRMZM2G079908 (ZmACBP1)

MGLQEEFEEHAEKAKTLPESTSNENKLILYGLYKQATVGDVNTDRPGIFYQKDRAKWDAWKAVEGKSKDEAMNDYITKVKQLQEEAAAS

> GRMZM2G344634 (ZmACBP2)

MGLQEEFEEHAEKAKTLPATTTNESKLVLYGLYKQATVGDVSTGRPGIFSLKERAKWDAW

KAVEGKSKDEAMADYITKVKQLLEAAAASTS

> GRMZM2G049495 (ZmACBP3)

MAGDWQELGQAAAIGLLFAFLVAKLISTVIAFKEDNLRITRSPPTSPTAAAAAAAPPVPS

HYGSTDGGSGSDSDWEGVESTELDEEFSAASAFIAASAASGTSVPEEAQLRLYGLYKIAT

EGPCTAPQPSALKLKARAKWNAWHKLGAMPTEEAMQGYITIVQDLFPNWDAGASAKRKDEDSIASASASKGPMGPVFSSLMYEEDEGNESELGDIHVSAREGATDDIVKLLAAGVEVNVRDTEGRSPLHWAVDRGHLSAVEVLANANADLNAKDNEGQTALHYAAVCEREDIAELLVKHHADLRIKDEDGNTAHDLCPSSWSFMNQAN

> GRMZM2G173636 (ZmACBP4)

MAGDWQDLGQAAAIGLLFAFLLAKLISTVIAFKEDNLRITRSPPSSPTAAAPAPATSPLP

SQHDAAIGSGSDSDWEGVESTELDEEFSAASAFVAASAASGTSVPEEAQLRLYGLYKIAT

EGPCTGPQPSALKLKARAKWNAWHKLGAMPTEEAMQEYITIVQELFPNWDAGTSAKRKDEESITSASASKGPMGPVFSSLMYEEDEGNDSELADIHVLAREGATEDIVKFLAAGVEVNMRDTEGRTPLHWAVDRGHLSAVEVLAKANADLNAKDNEGQTALHYAAVCEREDIAELLVKHHADLQIKDEDGNTAQDLCPPSWSFMNRAN

> GRMZM2G108138 (ZmACBP5)

MELFYELLLTAAVTLLAALLLATLFAANDSHRRTDRAAAAIAEEVAEEERIIEVDEVMRSEGMAAVAPSEADGWVEAEKAPAVVVAEVKEPESLPEEEGVPVKAVREVRLAAEVEEGEDGGGGVKRPDLTSATVVAAVGAEASQLVSGAEVVPKEVSGAAGLEERTVQDVVVNQYDLGAEVALVPVEVLEAGPDKQGVEVVGVAQVPPLETEAAEVKQHHLVAEAAPAEDVLDVGLVDKSVRAIEVRPNELDSETVLEEILDAVLEEEEQVEHELPAGAAPQPVLDVPLAGKGELKHHQPVEEAAEVHEDVQCKQEAECEAQTVDQQQELVPEEESVAGKNDDVNVIHECSFSDEVVTELPVGEVTSQGLPKDDPEADMEFEEWEGIERSEVEKRFGAAAAFAASGAGAAALSKLDSDVQLQLQGLLKVAIDGPCYDSAQPLTLRPSSRAKWSAWQKLGNMNPEIAMEKYMNLLSETIPGWMMNETLDTENTGSLPAETILTTMASTSDQRSNQGNEDNSSIGEGHPITSPNPEKGQSSDIPTE

> GRMZM2G060781 (ZmACBP6)

MAEWRNESEVRWNKVMNQFDLFVSDLVAAQFLGRATTSAYGEASYDRNTYCFNEEPVFDKGPIFYEEPVIDLELSSFNNVAPFNCSTYCLSQVIDVMTSKIASFDEGPLFDRYVIDEWPM

LDGELDCSTEPDEDRIPLKAARAMHPGAGLEENVQGLEVRLDELTALSQLNSDEHVQLQG

LLKVAIDDPCYDSTQPLTLRLSSHAKWSAWQKLGSMHPEIALEKYINLLSQVIPRWLRSE

IMDTKKYETKCYSVGFISRAATSDQQNYWESEDINSKRKDGRNMLLFWSTENVDRALEPYLMSNHNP

> GRMZM2G053803 (ZmACBP7)

MASSGLAYPDRFYAAAAYAGFGAGGATSSAAISRFQNDVALLLYGLHQQATVGPCNVPKP

RAWNPVEQSKWTSWHGLGSMPSAEAMRLFVKILEEEDPGWYSRVPEFNPEPVVDIQMHKPKDEPQSVPASTNGTSISEPKIISENGSSVETQDKDVILEGLSTVISHDEWTPLSVSGLRP

KPRYEHGATVLQNKMYIFGGNHNGRYLSDLQALDLKSLIWSKVDTKFQAEPADSTITTQI

APCAGHSLISWGNKFLSIAGHTKDPSEGITVKEFDPHTCTWSIVKTYGKPPVSRGGQTVT

LVGTTLVLFGGEDAKRCLLNDLHILDLETMTWDDVDAIGNPPSPRSDHAAACHADRYLLI

FGGGSHATCFNDLHVLDLQTMEWSRPKQQGLTPSPRAGHAGATVGENWYIVGGGNNKSGVSETLVLNMSTLTWSVVSTVEGRVPLASEGMTLVHSNYNGYDYLVSFGGYNGRYSNEVYTLKLSLKSDSQPIVEEETVSDTISRVREPETEISQDGRIREIAMDSADSDLNNRNDEASEQL

VADLKAQKEELEATINREQLQTVQLKEDIAQAETRNAELTKELQAVRGQLASEQSRCFKL

EVDVAELRQKLQSMDALEKEVELLRRQKAASEQAALDAKQRQGSGGMWGWLAGSPPPSQ

> Zm00001d012892 (ZmACBP8)

MGGKQLNIPRASATSAGSVKRRPRRDEASVGSPSPSLESLPPDLGASSPCRLTLQYIGEE

EELRFGRVNSTRRLAPVDRREGEIRAEIQGAAPAMASSGLAYPDRFYAAAAYAGFGVGEA

TSSAAISRFQNDVALLLYGLHQQATVGPCNVPKPRAWNPVEQSKWTSWHGLGSMPSAEAMRLFVKILEEEDPGWYSRAPEFNPEPVVDIQMHKPKDEPHSVPGSTNGTSIPEPNISENGS

SVEAQDKDVILEGLSTVSSHDQWTPLSVSGLRPKPRYEHGATVLQNKMYIFGGNHNGRYLSDLQALDLKSLTWSKVDAKLQAEPADSTKTTQIAPCAGHSLISWGNKFLSIAGHTKDPSE

GVTVKEFDPHTCTWSIVRTYGKPPVSRGGQTVTLVGTTLVLFGGEDAERCLLNDLHILDL

ETMTWDDVDAIGTPPSPRSDHAAACHADRYLLIFGGGSHATCFNDLHVLDLQTMEWSRPKQQGPTPSPRAGHAGGTVGENWYIVGGGNNKSVACDLLCIFMPNITHTLASTGVSETLVLNMSTLTWSVVSTVEGRVPLASEGMTLVHSNYGGYDYLISFGGYNGRYNNEVYTLKLSLKSDSQSTLKEETLSDTTSRVIEPEAEIYQDGKIREISVDNADSDLNNRNDEASEQLLADLKAQ

KEELEATLSREQLQTVQLKEDIARAETRNAELTKANHLSLTLNFKLSVANLLLSSQDVSN

LRYTLVDVAELRQKLQSMDALEKEVELLRRQRAASEQAAMDAKQRQSSGGMWGWLAGSPTPAV

> Zm00008a005731 (ZmACBP9)

MSPVPAAGWRPGAASGGRRGVWQATVGPCNVPKPRAWNPVEQSKWTSWHGLGSMPSAEAMRLFVKILEEEDPGWYSRVPEFNPEPVVDIQMHKPKDEPQSVPASTNGTSISEPKIISENGSSVETQDKDVILEGLSTVISHDEWTPLSVSGLRPKPRYEHGATVLQNKMYIFGGNHNGRYLSDLQALDLKSLIWSKVDTKFQAEPADSTITTQIAPCAGHSLISWGNKFLSIAGHTKDPS

EGITVKEFDPHTCTWSIVKTYGKPPVSRGGQTVTLVGTTLVLFGGEDAKRCLLNDLHILD

LETMTWDDVDAIGNPPSPRSDHAAACHADRYLLIFGGGSHATCFNDLHVLDLQTMEWSRPKQQGLTPSPRAGHAGATVGENWYIVGGGNNKSGVSETLVLNMSTLTWSVVSTVEGRVPLASEGMTLVHSNYNGYDYLVSFGGYNGRYSNEVYTLKLSLKSDSQPIVEEETVSDTISRVREPETEISQDGRIREIAMDSADSDLNNRNDEASEQLVADLKAQKEELEATINREQLQTVQLK

EDIAQAETRNAELTKASSLTLFLLELQAVRGQLASEQSRCFKLEVDVAELRQKLQSMDAL

EKEVELLRRQKAASEQAALDAKQRQGSGGMWGWLAGSPPPSQ

> OS08G06550 (OsACBP1)

MGLQEDFEQYAEKAKTLPESTSNENKLILYGLYKQATVGDVNTARPGIFAQRDRAKWDAWKAVEGKSKEEAMSDYITKVKQLLEEAAAAAS

> OS06G02490 (OsACBP2)

MGLQEEFEEFAEKAKTLPDTISNEDKLLLYGLYKQATVGPVTTGRPGIFNLKDRYKWDAW

KAVEGKSKEEAMADYITKVKQLLEEASASTS

> OS03G37960 (OsACBP3)

MGLQEDFEEYAEKVKTLPESTSNEDKLILYGLYKQATVGDVNTSRPGIFAQRDRAKWDAWKAVEGKSKEEAMSDYITKVKQLQEEAAALKAVFRAYLVGEMNIFECHIGRLTRCRRGFRTQMKKQIVYSPGTREMNLLSLIKPSLAHVGYCSTYG

> OS04G58550 (OsACBP4)

MGGDWQELAQAAVIGLLFAFLVAKLISTVIAFKEDNLRITRSTPTSPSAADTPAAPAPPP

ASLDGGHGDTSDGSGSDSDSDWEGVESTELDEEFSAASAFVAASAASGTSVPEQAQLQLY

GLYKIATEGPCTAPQPSALKLKARAKWNAWHKLGAMPTEEAMQKYITVVDELFPNWSMGSSTKRKDEDTTVSASSSKGPMGPVFSSLMYEEEDQGNDSELGDIHVSAREGAIDDIAKHLAAGVEVNMRDSEGRTPLHWAVDRGHLNSVEILVNANADVNAQDNEGQTALHYAVLCEREDIAELLVKHHADVQIKDEDGNTVRELCPSSWSFMNLAN

> OS03G14000 (OsACBP5)

MELFYELLLTAAASLLVAFLLARLLASAATASDPRRRAPDHAAVIAEEEAVVVEEERIIE

VDEVEVKSARARECVVSEGWVEVGRASSAEGKLECLPEEEEAPAKAARELVLDAVLEEREEEGQVGEERCDLAAAVAEVVGVKPHELGVEAAPGEVSDVTLEEGKVQDVGVEQHDLVAEAAPREALDTGLEKQGVPIIEAVEIKRQDDLGAEVAPSDVPEVEFEQQGVRIIEAIDVNQHHRVALAAPAEVVDAGLEERVQAIEAGSSGLTSETVPEEVLDELSEKQEEQVIEEKEHQLAA

ATAPVAIPGVALAETEELKEEQSSEKAVNVHEEVQSKDEAKCKLHLVDQQEGSASKVELV

GRNTDNVEISHGSSSGDKMIAELTEEELTLQGVPADETQTDMEFGEWEGIERTEIEKRFG

VAAAFASSDAGMAALSKLDSDVQLQLQGLLKVAIDGPCYDSTQPLTLRPSSRAKWAAWQKLGNMYPETAMERYMNLLSEAIPGWMGDNISGTKEHEAGDDAVGSVLTMTSNTINQHDSQGNEDNTGMYEGHLTSSPNPEKGQSSDIPAE

> OS03G61930 (OsACBP6)

MASSGLAYPDRFYAAAAYAGFGAGGATSSSAISRFQNDVALLLYGLYQQATVGPCNVPKP

RAWNPVEQSKWTSWHGLGSMPSAEAMRLFVKILEEEDPGWYSRVPEFNPEPVVDIEMHKPKEDPKVILASTNGTSVPEPKTISENGSSVETQDKVVILEGLSAVSVHEEWTPLSVNGQRP

KPRYEHGATVVQDKMYIFGGNHNGRYLSDLQALDLKSLTWSKIDAKFQAGSTDSSKSAQVSSCAGHSLISWGNKFFSVAGHTKDPSENITVKEFDPHTCTWSIVKTYGKPPVSRGGQSVT

LVGTTLVLFGGEDAKRCLLNDLHILDLETMTWDDVDAIGTPPPRSDHAAACHADRYLLIF

GGGSHATCFNDLHVLDLQTMEWSRPKQQGLAPSPRAGHAGATVGENWYIVGGGNNKSGVSETLVLNMSTLTWSVVSSVEGRVPLASEGMTLVHSNYNGDDYLISFGGYNGRYSNEVFALKLTLKSDLQSKTKEHASDGTSSVLEPEVELSHDGKIREIAMDSADSDLKDDANELLVALKAEKEELEAALNREQVQTIQLKEEIAEAEARNAELTKELQTVRGQLAAEQSRCFKLEVDVAELRQKLQSMDALEREVELLRRQKAASEQAALEAKQRQSSSGMWGWLVGTPPDKSES

> Bradi3g16180 (BdACBP1)

MGLKEEFEEHAEKAKTLPETTSNENKLILYGLYKQATVGVVNTPRPGLFNLKDKAKWDAWKAVEGKSTEEAMSDYITKVKQLQEEASAA

> Bradi1g50540 (BdACBP2)

MGLQEEFEEHAEKAKTLPDTTTNENKLLLYGLYKQATVGPVNTARPGFFDLKGKAKWDAWKVLEGKSKEEAMTDYITKVKQLLEEATAAAASTS

> Bradi5g26460 (BdACBP3)

MGGDWQELAQAAVIGLLFAFFAAKLISVVVAFKDDNLRITRSPPAVPSPSPADPPPPPAS

LDGGVGSGSDSDGDWEGVESTELDEDFSAASAFVAASAASGTSVPEEAQLRLYGLYKIAT

EGPCTAPQPSALKLKARAKWNAWNKLGAMPTEEAMEEYITIVDDLFPNWADGSSAKRKDGDSTMSASGSKGPMGPVFSSLMYEEDQGNESELGDIHVSAREGAIDDVKKHLAAGVEVNIRDSEERTPLHWAVDRGHLSAVEVLVNSNADVNAQDNEGQTALHYAVLCEREDIAELLVKHHADLQIKDGDGNTPQDLCSSTAWSFMNPAN

> Bradi1g68300 (BdACBP4)

MELFFELLLTAAASLLVAFLLAKLFSANDPRSDPRDRAVGPADVIAEGNEEEEQERIIEV

DEVKVKRAWADVAAPTLAEEWVEVEKAPATVAEEKTRCLPEEVGIPARLAPELFLGAVLE

GRKEEGEVGKKPCDLTSAAAAMETSVEVKLRDLGAESSPSPREVIDVELEKEGEQQHDLC

AEVAPCEVLDAGSQKQEVQAIEAVEVEQRHLAAPKEVIDAALAQECSQTLAEIPHELASD

AVPDEVLEAVFEKQEQQVIEVNQQELTSEVAPRVPVDVALAEKDELQDNPVEEVVDVHEE

AQSDDKAKCDASMVGRQTELVPMEDLVVMKDDDPEVSHDGSSNDKVAVQLPEKEVTLLGMPEDETRACMEFEEWEGIERSEVEKRFGAAAAFSASDAGTAALSKLDSDVQLRLQGLLKVAIDGPCYDSTQPLTLRPSSRAKWVAWQKLGNMHPEIAMDKYMNLLSEIIPGWMGDKTNSSIKKHEADGDSEEPVLAMTDHKGDQHIYQRNEDSTSTSMDEGPLPSPPNPEKGQSSDVPAE

> Bradi1g02597 (BdACBP5)

MASAGIAYPDRFYAAAAYAGFGAYGATTSSSAAISRFQNDVALLLYGLHQQATVGPCNVP

KPRAWNPVEQSKWTSWHGLGSMPQAEAMRLFVKILEEEDPGWYSRVPELNPRPVVDIEMLKPKEEPNVLPASTNGTTPIPEPKTISENGSSVETQDKVVILEGLSTVSAHEEWTALSPSG

QRPKPRYEHGATVLQDKMYIFGGNHNGRYLSDLQVLDLKSLSWSKIDAKLQAGSSDLAKTTQVSPCAGHSLISWGNRFFSIAGHTKDPSDNVTVKEFDPHTCTWSIVSTYGKPPVSRGGQ

SVTLVGTTLVVFGGEDAKRCLLNDLHILDLESMTWDDVDAIGTPPAPRSDHAAACHADRYLLIFGGGSHATCFNDLHVLDLQTMEWSRPKQQGLLPSPRAGHAGATVGENWYIVGGGNNKSGVSETLVLNMSTLAWSVVSTVEGRVPLASEGMTLVCSNYSGEDYLISFGGYNGRYSNEVYALKLSVKSDLQSNTEDQPISDSTSRVLEPEVEISQDGKIREIAMDNSDSVNRNDEASEQ

LLAALKAEKEELEATLNREGLQTVQLKEEITEAEARNAELTKELQAVRGQLAAEQSRCFK

LEVDVAELRQKLQSLDALEREVELLRRQRAASEQAALDAKQKQGSGGVWGWLVGTPPDDSES

> Traes_4AL_7B8E7660A (TaACBP1)

MGLKEDFEEYAEKAKALPETQSTSNEDKLILYGLFKHATVGVANTARPGMCNMRERAKWDAWEAVKDKSKEEAMNDYITKVKQLQEEAAAAGAC

> Traes_7DL_6B7B8EE08 (TaACBP2)

MGLQEEFEEYAEKAKTLPDTTTNESMLCLYSLYKQATVGPVNTARPGMFDLKGKAKWDAWKSVEAKSKEEAMADYITKVKQLLEEAAAASASS

> Traes_7DS_595FB94A0 (TaACBP3)

MLCLPADVVQEEFEEHAEKAKTLPDTTTNESKLCLYGLYKQATVGPVNTARPGGLFDMAGKAKWDAWKAVEAKSKEEAMADYITKVKQLLEEAAAASA

> Traes_7AS_AD936D17A (TaACBP4)

MPRLPIDVVQEEFEEHAEKAKTLPDTTTNESKLCLYGLYKQATVGPVNTARPGGLFDMAG

KAKWDAWKAVEAKSKEEAMADYITKVKQLLEEAAAASA

> Traes_2AL_E2F5A6BD2 (TaACBP5)

MGGDWQELAQSAVIGLIFAFLVAKLISVVLAFKEDNLRITRSAPDPAPSSSPDAAAPALD

GGDGDGDSDSDGDWEGVESTELDEDFSAASAFVAASAAGVPEEAQLRLYGLYKIATEGPC

TAPQPSALKLKARAKWNAWNKLGAMPTEEAMEEYITIVDEIFPNWSDGSKKKDGETTMSASGSKGPMGPVFSSLMYEEDQGNESELGDIHVSAREGAIDDVKKHLAAGVQINIRDSEERTPLHWAVDRGHLDAVEVLVNSNADVNAQDNEGQTALHYAVLCEREDIAELLVKHHADLQIKDGDGNTAQDLCSSAWPFMKPAN

> Traes_2BL_4157B2613 (TaACBP6)

MGGDWQELAQSAVIGLIFAFLVAKLISVVLAFKEDNLRITRSAPAPAPSPDATAPALDGG

TSGGDGDSDGDWEGVESTELDEDFSAASAFVAASAAGVPEEAQLRLYGLYKIATEGPCTA

PQPSALKLKARAKWNAWNKLGAMPTEEAMEEYITIVDEIFPNWADGSKKKDGETTMSASGSKGPMGPVFSSLMYEEDQGNESELGDIHVSAREGAIEDVKKHLAAGVQINLRDSEERTPLHWAVDRGHLDAVEVLVNSNADVNAQDNEGQTALHYAVLCEREDIAELLVKHHADLQIKDGDGNTARDLCSSAWPFMKPAN

> Traes_4AS_312C47A21 (TaACBP7)

MTEWVAAEEEGVPATLLAPELFLGTVSREQKEEGEVGKKHCDLTAAAEAAVEVKPRDSGDEAAPREVLGVELEEETTQQRNDLGAEVAPSEVPDAGLQKQEVHAMEAVEVKQLHLDVGAAPAEVIDAGPEEREEGVQAAEVIPRELAPETLPSDVLDVIPGKQEEQVIEASQHELPLVVAPRVIPDAAAKDEEMKEQSVEEVVVPQEEVHSKEEAQCEAGRDDQHEELVPKDEPALKKSDDLSVSQEDSPNDKVDVQLPEKDTTLLGMPEDEARASMEFEEWEGIERSEVEKRFGAAAAFAASDTGAAALSKLNTDVQLQLQGLLKVAVDGPCYDAAQPLTLRPSSRAKWVSWQKLGNMHPEIAMEKYMNLLSEFIPGWMGDATSSTEKHKVDVDSEGALLTMTTHTSDPQINQGNEGSASIDEGPLTSPPNPEIGQSSDVPAE

> Traes_4DL_B62D219D8 (TaACBP8)

MEAVEVKQLHLDVGAAPAEVIDAGPEEKAEGVQAAEVIPRELASETPPADVLDVVIGKQE

EQVIEASQHELAPVVAPRVIPDAAAKDEEMKEQSVEEVVQVHNKEEARREAGTDDQQEKLVPKDEPALEKSDDLNVSQEDSSNDKVDVQLPEKDTTLLGMPEDEARASMEFEEWEGIERSEVEKRFGAAAAFAASDAGAAALSKLNSDVQLQLQGLLKVAVDGPCYDAAQPLTLRPSSRAKWVSWQKLGNMHPEIAMEKYMNLLSEFVPGWMGDTTPSTEKHKVDVDSEGAVLTMTTHTSDPQISQGNEGSTSIDEGPLTSPPNPEKGQSSDVPAE

> Traes_4AL_3AE0F6921 (TaACBP9)

MASSGIAYPDRFYAAAAYAGFGAGAPSTAAISRFQNDVALLLYGLHQQATVGPCNVPKPR

AWSPVEQSKWTSWHGLGSMPSAEAMRLFVKILEEEDPGWYSRIPEFINPQPVVDIEMHKP

KEEPDIVPALTNGTGTSSIPEPKTISESGSSVETQDKVVILEGLSTVSAHEEWTALSVSG

QRPKPRYEHGATVLQDKMYIFGGNHNGRYLSDLQVLDLKSLTWSKVDAKLQAGTSDSAKTAQVSPCAGHSLISCGNKFFSVAGHTKDPSDSITVKEFDPHTCTWSIVKTYGKPPVSRGGQSVTLVGTTLVVFGGEDAKRCLLNDLHILDLETMTWDDVDAIGTPPAPRSDHVAACHADRYLLIFGGGSHATCFNDLHVLDLQTMEWSRPKQQGPIPSPRAGHAGATVGENWYIVGGGNNKSGVSETLVLNMSTLAWSVVSTVEGRVPLASEGMTLLYSNYSGEDYLISFGGYNGRYSNEVYALKLSVKLDLQSSTKDQPTSDSTSRVLEPEVEISQDGKIREIAMDNADSKNRNDEANEQLLAALKAEKDELEATLNREGLQTVQLKEEITEAEAXXXXXXXELQAVRGQLAAEQSRCFKLEVDVAELRQKLQSLDALEREVELLHRQKAASEQEQAALDAKQKKQAGSGGVWGWLVGTPPDDDDSESS

> Traes_5BL_CBFC251AA (TaACBP10)

MASSGIAYPDRFYAAAAYAGFGAGAPSTAAISRFQNDVALLLYGLHQQATVGPCNVPKPR

AWSPVEQSKWTSWHGLGSMPSAEAMRLFVKILEEEDPGWYSRIPEFINPQPVVDIEMHKP

KEEPDIVPALTNGTGTSPIPEPKTISENGSSVETQDKVVILEGLSTVSAHEEWTALSVSG

QRPKPRYEHGATVLQDKMYIFGGNHNGRYLSDLQVLDLKSLTWSKIDAKLQAGTSDSAKTAQVSPCAGHSLISCGNKFFSVAGHTKDPSDSITVKEFDPHTCTWSIVKAYGKPPVSRGGQ

SVTLVGTTLVVFGGEDAKRCLLNDLHILDLETMTWDDVDAIGTPPAPRSDHVAACHADRY

LLIFGGGSHATCFNDLHVLDLQTMEWSRPKQQGPIPSPRAGHAGATVGENWYIVGGGNNKSVSETLVLNMSTLAWSVVSTVEGRVPLASEGMTLLYSNYSGEDYLISFGGYNGRYSNEVYALKLSVKLDLQSSTKDQPTSDSTSRVLEPEVEISQDGKIREIAMDNADSKNRNDEANEQL

LAALKAEKDELEATLNREGLQTVQLKEEITEAEARNAELTKELQAVRGQLAAEQSRCFKL

EVDVAELRQKLQSLDALEREVELLRRQKAASEQEQAALDAKQKKQAGSGGVWGWLVGTPP

DDDDSESS

> Traes_5DL_3CE28EBD4 (TaACBP11)

MAGIRDREFGLVATDLVVFVSAQATVGPCNVPKPRAWSPVGQSKWTSWHGLGSMPSAEAMRLFVKILEEEDPGWYSRIPEFINPQPVVDIEMHKPKEEPDIVPLTNGTGTSSIPEPKTIS

ENGSSVETQDKVVILEGLSTVSAHEEWTALSVSGQRPKPRYEHGATVLQDKMYIFGGNHN

GRYLSDLQVLDLKSLTWSKIDAKLQAGTSDSAKTAQVSPCAGHSLISCGNKFFSVAGHTK

DPSDSITVKEFDPHTCTWSIVKTYGKPPVSRGGQSVTLVGTTLVVFGGEDAKRCLLNDLH

ILDLETMTWDDVDAIGTPPAPRSDHVAACHADRYLLIFGGGSHATCFNDLHVLDLQTMEWSRPKQQGPIPSPRAGHAGATVGENWYIVGGGNNKSGVSETLVLNMSTLAWSVVSTVEGRVPLASEGMTLLYSNYSGEDYLISFGGYNGRYSNEVYALKLSVKLDLQSSTKDQPTSDSTSRVLEPEVEISQDGKIREIAMDNADSKNRNDEANEQLLAALKAEKDELEATLNREGLQTVQLKEEITEAEARNAELTKELQAVRGQLAAEQSRCFKLEVDVAELRQKLQSLDALEREVELLRRQKAASEQEQAALDAKQKKQAGSGGVWGWLVGTPPDDDDSESS
