# Supplementary material for: Molecular characterization, expression and functional analysis of acyl-CoA-binding protein gene family in maize (Zea mays)
Source: BMC Plant Biol. 2021 Feb 15;21:94. doi: 10.1186/s12870-021-02863-4 (PMC7883581; doi:10.1186/s12870-021-02863-4)
Supplement: Supplementary file 8 — Additional file 8: Table S5–6. Primers and their sequences used in this study. [file 12870_2021_2863_MOESM8_ESM.docx]

Table S5 Primer sequences used to isolate *ZmACBPs* cDNAs.

| gene | Primer name | Primer sequence |
| --- | --- | --- |
| *ZmACBP1* | F  R | CGGAGGAGAAACAAAAGACGC  TATGCGACTGCGGATCATGG |
| *ZmACBP2* | F  R | GGCCCGAACCTGTAAACACT  TTCTGGATCCCATTGCCACA |
| *ZmACBP3* | F  R | CCTTTCTTCCTTCCCCCAGAT  TCAGAGAGGGCACCACGTA |
| *ZmACBP4* | F  R | CGGGCCCTACCAGTTTCG  CCAAGTGCGGTACCAGCTTA |
| *ZmACBP5* | F  R | AGAAAGCGAAGGGGCCAAAA  CAACCGCCACAACTGTACAAA |
| *ZmACBP6* | F  R | CCATTTTGCTACAGTCGGCG  AAGCCCATCGCCTGAACATT |
| *ZmACBP7* | F  R | TGGACGATACAGCAACGA  CAGGTCTGAATCGGCACT |
| *ZmACBP8* | F  R | AATCCTAACAGCCGTCTCGC  TTAGTCGAAGTGGCTGTGCC |

Table S6 The primers used for real-time quantitative RT-PCR (qRT-PCR)

| gene | Primer name | Primer sequence |
| --- | --- | --- |
| *ZmACBP1* | F  R | ATCCTCTACGGGCTCTACAA  TCATTCATCGCCTCATCC |
| *ZmACBP2* | F  R | CCACCACGACGAACGAGA  CAGCCTTCCAAGCATCCC |
| *ZmACBP3* | F  R | GGCGACTGATGACATAGTGAAG  AGCATAGTGGAGTGCCGTTT |
| *ZmACBP4* | F  R | TCCACTACGCTGCTGTTTGT  CTGTGCCGTGTTCCCATC |
| *ZmACBP5* | F  R | GGCAGAATGGAGGAATGA  ATATGCTGATGTCGTGGC |
| *ZmACBP6* | F  R | TGAAGTGAAACAGCACCATCT  TCCAATACCGCATCAAGAAT |
| *ZmACBP7* | F  R | TGGACGATACAGCAACGA  CAGGTCTGAATCGGCACT |
| *ZmACBP8* | F  R | CTGCGGAAGGACGAGTT  TCTGATTTGAGGCTAAGTGT |
| *ZmACBP9* | F  R | TGGACGATACAGCAACGA  CAGGTCTGAATCGGCACT |
| *ZmTUB* | F  R | CTACCTCACGGCATCTGCTATGT  GTCACACACACTCGACTTCACG |
| *AtRD29A* | F  R | GACGAGTCAGGAGCTGAGCTG  CGATGCTGCCTTCTCGGTAGAG |
| *AtRD29B* | F  R | CCGACGGGAACTCATGATCAGTTC  CACTTCCACCTCCTTTGTAGCCG |
| *AtCOR47* | F  R | GAAGCTCCCAGGACACCACGAC  CAGCGAATGTCCCACTCCCAC |
| *AtRAB* | F  R | CGTCTTACCAGAACCGTCCA  CCGTAGCCACCAGCATCATA |
| *AtABI1* | F  R | GCTCTGCGATGGTGATAC  GTTAGCGACGAAGATGTGA |
| *AtAREB1* | F  R | AGGCGTTGGTGCTGTG  GCTTTCGTTGTAACTCGTC |
| *AtFAD2* | F  R | CGCTCTATCTTTCGTCTTCGG  CGTCTTTGGTTCTCTGTAGTATCTTC |
| *AtAPLDα1* | F  R | TGTGCCCTCGTAACCC  GGAACGGAGTGTCGTATCTT |
| *AtPLC3* | F  R | CAAGGACATGGGAAGCAACT  CTTTTGCAAGGGTCGAAGAG |
| *AtACX* | F  R | TGAAGGAGCAACTGTGCATC  GGGTAAACATTGCCGTCGTA |
| *AtDGAT* | F  R | GCAGCAAGATACCAAAGACA  CCTGAAACATAATCCCAAGAA |
| *AtPLA2* | F  R | ATGATCCGCGGTGGTGCTTTGACAC  GTGTCAAAGCACCACCGCGGATCAT |
| *AtActin* | F  R | CCCGCTATGTATGTCGC  AAGGTCAAGACGGAGGAT |
